# Revival of Layered Ferroelectrics in Thin Films

**DOI:** 10.1002/smsc.202500488

**Published:** 2026-01-24

**Authors:** Elzbieta Gradauskaite

**Affiliations:** ^1^ Laboratoire Albert Fert, CNRS, Thales Université Paris Saclay 91767 Palaiseau France

**Keywords:** Aurvillius, Carpy–Galy, ferroelectric thin films, layered perovskites, Ruddlesden–Popper

## Abstract

Layered perovskites form a versatile class of ferroelectrics in which structural anisotropy gives rise to periodic electrostatics and, consequently, unconventional ferroelectric properties. These materials fall into four main families: Aurivillius, Carpy–Galy, Ruddlesden–Popper, and Dion–Jacobson phases, each forming natural superlattices by interleaving perovskite slabs with spacer layers. For a long time, these materials were considered too structurally complex to prepare as high‐quality thin films. However, recent breakthroughs in deposition and advanced characterization have made it possible to stabilize epitaxial films with atomic‐scale control, uncovering novel ferroelectric functionalities. These include robust in‐plane polarization without a critical thickness, the emergence of charged domain walls and non‐trivial polar textures, resilience to doping with magnetic ions and charge carriers, and the possibility to epitaxially integrate them into standard perovskite heterostructures. This review aims to unify current knowledge on the fabrication and characterization of layered ferroelectric thin films, and to present research findings across all four structural families, with the goal of highlighting their common features despite differences in crystal structure and polarization mechanisms. We also discuss promising research directions, including polar metallicity, (alter‐)magnetoelectricity, exfoliation, and soft‐chemistry‐driven phase transformations, hoping to encourage exploration of these materials for both fundamental studies and applications.

## Graphical abstract

This review presents recent advances in layered perovskite‐based ferroelectric thin films, showing how progress in deposition and characterization now enables atomic‐precision synthesis across the Aurivillius, Carpy–Galy, Ruddlesden–Popper, and Dion–Jacobson families. It unifies current understanding of their robust in‐plane polarization, unconventional polar textures, and emerging functionalities, including polar metallicity, (alter‐)magnetoelectricity, and soft‐chemistry‐driven phase transformations.

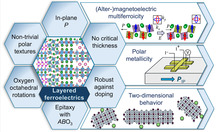

## 1 Introduction

Complex perovskite oxides can enable a broad range of physical phenomena, from magnetism^[^ ^1^, ^2^, ^3^ ^]^ and ferroelectricity^[^ ^4^, ^5^ ^]^ to multiferroicity,^[^ ^6^, ^7^, ^8^ ^]^ superconductivity,^[^ ^9^, ^10^ ^]^ and catalytic activity.^[^ ^11^ ^]^ This functional diversity is underpinned by the chemical flexibility of the perovskite structure (*AB*O_3_), which can accommodate a broad range of cations on both the *A* and *B* sites.^[^ ^12^ ^]^ The perovskite structure consists of a network of corner‐sharing *B*O_6_ octahedra, with the larger *A*‐site cation at the corners of the (pseudo‐)cubic unit cell. This simple yet versatile framework can easily distort, tilt, and adapt to nonstoichiometries, providing a powerful handle for tuning material properties. Beyond structural versatility, the richness of physical properties in perovskite oxides stems from the electronic configuration of the transition metal cations, particularly the spatially localized *d*‐ and *f*‐orbitals.

A breakthrough for oxide interface physics in the early 2000s was the realization that atomically sharp interfaces between oxide perovskites can give rise to emergent phenomena absent in the bulk. The most iconic example is the discovery of conductivity at the interface between two insulating oxides, LaAlO_3_ and SrTiO_3_.^[^ ^13^ ^]^ Other examples include charge transfer effects^[^ ^14^ ^]^ and interfacial magnetoelectric coupling^[^ ^15^, ^16^, ^17^ ^]^ mediated by strain or charge coupling. These discoveries triggered a surge of interest in artificially constructed superlattices, where the interfacial effects are maximized relative to the bulk and dominate the overall physical behavior.

Superlattice design has proven particularly fruitful in the field of ferroelectrics. By precisely tuning the periodicity (or the “superlattice wavelength”) of alternating ferroelectric and dielectric layers (e.g., PbTiO_3_ and SrTiO_3_, respectively), researchers have achieved control over the ferroelectric state.^[^ ^18^ ^]^ The PbTiO_3_ system, known for its single‐domain out‐of‐plane polarization in epitaxial thin films,^[^ ^19^ ^]^ evolves with increasing periodicity through multidomain out‐of‐plane states^[^ ^20^ ^]^ to fully in‐plane‐polarized configurations.^[^ ^21^ ^]^ The delicate balance between elastic, electrostatic, and gradient energies can even stabilize hybrid improper ferroelectricity^[^ ^22^ ^]^ or nontrivial ferroelectric topologies such as flux‐closure patterns,^[^ ^23^ ^]^ polar vortices,^[^ ^24^, ^25^ ^]^ 3D supercrystals,^[^ ^26^ ^]^ and antipolar order.^[^ ^27^ ^]^ All this is governed by the interplay between strain and electrostatic boundary conditions at each interface with dielectric layers.

Nature provides an alternative route to such layered oxide architectures in the form of “natural superlattices”: crystal structures that exhibit periodic stacking driven purely by inherent bonding preferences and unit‐cell electroneutrality, without the need for artificial layering or externally imposed strain. Several families of such layered perovskite‐based oxides exist, including Aurivillius (A), Carpy–Galy (CG), Ruddlesden–Popper (RP), and Dion–Jacobson (DJ) phases. These compounds are composed of a variable number *n* of perovskite‐like planes interleaved with ionically distinct spacer layers along one crystallographic direction. This results in large unit cells with pronounced anisotropy, which often gives rise to emergent phenomena not seen in their perovskite counterparts. While most renowned for the superconductivity observed in the Ruddlesden–Popper phases,^[^ ^28^ ^]^ many of these layered compounds are also ferroelectric and exhibit a sizeable in‐plane polarization.^[^ ^29^ ^]^

These naturally layered compounds were intensively studied in the 1990s for their fatigue‐free polarization switching.^[^ ^30^ ^]^ Their integration into devices was nevertheless slowed by challenging synthesis: layered perovskites were mostly available as polycrystalline ceramics, and their structural complexity made it difficult to achieve well‐oriented and defect‐free films. Over the past decade, however, interest in these compounds has revived, driven by major advances in epitaxial stabilization of single‐crystalline films, in situ growth monitoring, and modern characterization techniques, which now enable the synthesis of single‐crystalline thin films and the exploration of functionalities that were inaccessible in bulk form.

**Figure 1:**
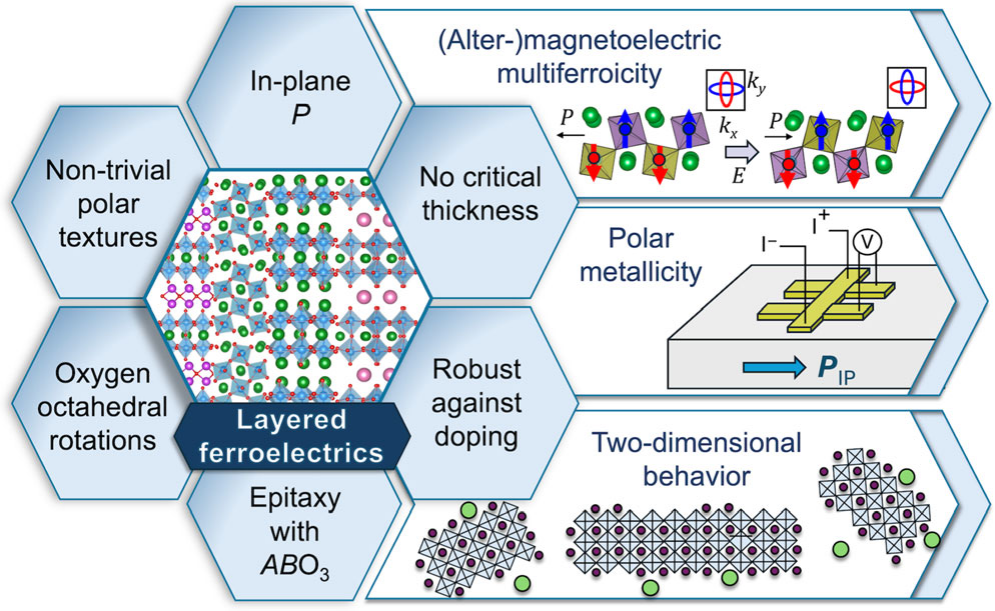
Graphical outline of the review: the unique properties and future potential applications of layered ferroelectric thin films. The hexagonal panels highlight characteristic properties of layered perovskite‐based ferroelectric oxides, particularly in thin‐film form, which are discussed in Section 5. The chevron panels indicate three key research opportunities discussed in this review, Section 6.

This review aims to contextualize the revival of interest in layered perovskite‐based ferroelectric oxides, with particular emphasis on recent experimental progress in thin‐film synthesis and characterization. Complementary to earlier reviews that focused on theoretical polarization mechanisms in layered perovskites^[^ ^29^ ^]^ and 2D ferroelectrics,^[^ ^31^, ^32^ ^]^ this review aims to connect recent experimental and theoretical progress across all four oxide‐based layered perovskite families. **Figure** **1** serves as a visual guide to the review's perspective on the unique properties and future potential of these materials. We begin by introducing the structural families and their associated polarization mechanisms (Section 2). We then trace the evolution of thin‐film growth strategies that enabled better crystalline quality and control over orientation in Section 3. Characterization approaches specific to these materials are discussed in Section 4, including defect analysis and its implications for ferroelectric functionality. This is followed by a survey of unconventional phenomena in layered ferroelectrics that emerge in the (epitaxial) thin‐film limit (Section 5). We conclude with a discussion on key opportunities (Section 6), ranging from polar metallicity and multiferroicity to soft‐chemistry methods for structural tuning, outlining exciting avenues for future research in layered perovskite‐based ferroelectric oxides.

## 2 Structural Families and Associated Mechanisms for Ferroelectricity

There are four main families of layered perovskite‐based ferroelectrics: Aurivillius, Carpy–Galy, Ruddlesden–Popper, and Dion–Jacobson. The Aurivillius ferroelectrics (also referred to as bismuth layered ferroelectrics (BLFs)) were first reported in 1949^[^ ^33^ ^]^ and are by far the most studied, with the interest in these materials peaking in the 1990s due to their fatigue‐free properties explored in SrBi_2_Ta_2_O_9_ capacitors.^[^ ^30^ ^]^ The Carpy–Galy family with La_2_Ti_2_O_7_ as a prototypical example has been studied significantly less, even though ferroelectricity in these compounds was uncovered back in the 1970s.^[^ ^34^, ^35^ ^]^ One reason for that could be the lack of a clear unifying family name, as these materials were often referred to as perovskite‐like layered structures (PLS) or simply as (110) layered ferroelectrics. It was not until 2019 that Núñez Valdez and Spaldin^[^ ^36^ ^]^ insisted on the Carpy–Galy name, which will hopefully help to unify research efforts on this phase and render them more accessible.

While Aurivillius and Carpy–Galy materials can be classified as proper ferroelectrics, ferroelectricity in Ruddlesden–Popper^[^ ^37^ ^]^ and Dion–Jacobson^[^ ^38^, ^39^ ^]^ phases is typically hybrid improper, i.e., two nonpolar distortions are the primary order parameters that induce ferroelectric polarization through trilinear coupling. Initially predicted from first‐principles calculations in 2011 by Benedek and Fennie,^[^ ^40^ ^]^ hybrid improper ferroelectricity was first experimentally demonstrated in Ruddlesden–Popper compounds such as Ca_3_Mn_2_O_7_ in 2015.^[^ ^41^ ^]^ Ferroelectricity in Dion–Jacobson compounds, such as RbNdNb_2_O_7_, was theoretically predicted in 2006,^[^ ^42^ ^]^ experimentally confirmed in 2012,^[^ ^43^ ^]^ and later explained by the unifying theory of hybrid improper ferroelectricity.^[^ ^44^ ^]^ Hybrid–improper ferroelectricity has likewise been predicted for *A*‐site–ordered double perovskites, (*A*,*A’*)*B*_2_O_6_.^[^ ^29^, ^45^ ^]^ Because such cation‐ordered structures are rarely found in nature, they are not discussed further in this review.

We provide a brief overview of the structural differences between the four families, along with a short description of the polarization mechanisms characteristic of each. For a more detailed discussion of the crystal‐chemical origins of polarization in these materials, we refer the reader to the seminal review by Benedek et al.^[^ ^29^ ^]^

### 2.1 Aurivillius Bi_2_O_2_[*A*_*n*−1_ *B*_*n*_O_3*n*+1_] Compounds

**Figure 2:**
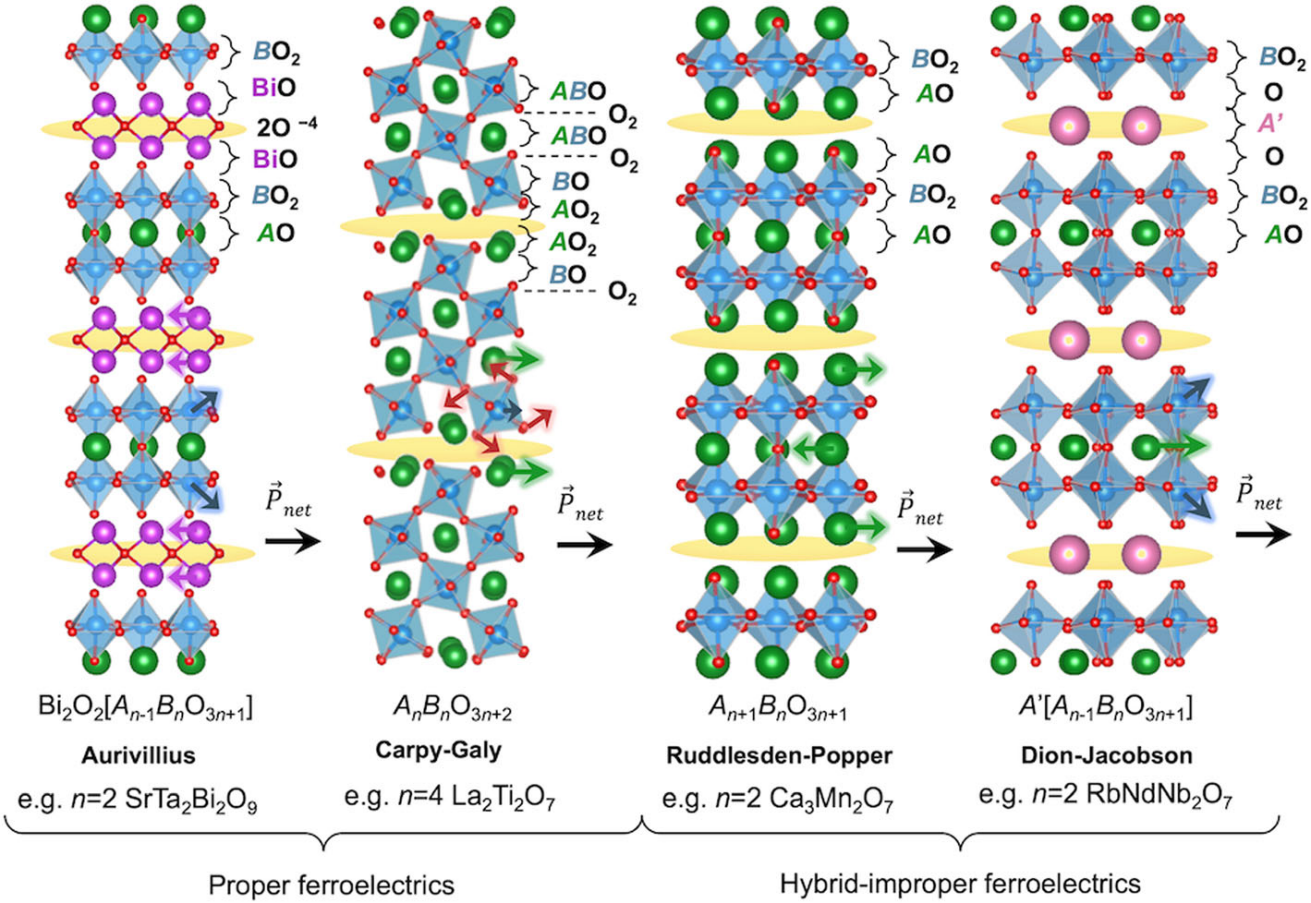
Structural families of layered perovskite‐based ferroelectric oxides. Schematic representations of four families: Aurivillius, Carpy–Galy, Ruddlesden–Popper, and Dion–Jacobson. Yellow highlights the additional spacer layers, which concentrate charge and influence the polarization mechanisms. Colored arrows indicate typical ionic displacements driving ferroelectricity in each structure. Atomic plane labels on the right side denote the stacking sequence within each family.

The unit cell of Aurivillius compounds^[^ ^33^, ^46^ ^]^ is made up of alternating layers of a variable number *n* of perovskite‐octahedra planes interleaved between fluorite‐like [Bi_2_O_2_]^2+^ layers along the *c*‐axis, see **Figure** **2**. Their high‐symmetry phase is a tetragonal *I*4/*mmm* structure that undergoes one or more^[^ ^47^ ^]^ transitions to a polar phase (e.g., orthorhombic *A*2_1_ *am*) upon cooling, characterized by large spontaneous in‐plane polarization (30–50 μC cm^−2^).^[^ ^46^ ^]^

The proper ferroelectricity in Aurivillius phases originates from in‐plane distortions, where the [Bi_2_O_2_]^2+^ fluorite‐like layers shear relative to the perovskite blocks.^[^ ^46^, ^48^, ^49^ ^]^ In addition to the ferroelectric soft mode, Aurivillius compounds exhibit additional nonpolar distortions, such as in‐plane tilts and out‐of‐plane rotations of the oxygen octahedra.^[^ ^29^, ^49^ ^]^ Bi^3+^ cations exhibit lone‐pair‐driven off‐center displacements along the crystallographic *a*‐axis (the in‐plane polar axis), indicated by purple arrows in Figure 2. Within each perovskite slab, the oxygen octahedra tilt and rotate, and the *B* cations shift off‐center (blue arrows in Figure 2). These *B*‐site off‐centerings adopt an antipolar‐like order along the out‐of‐plane direction, while their in‐plane components add up to produce net in‐plane polarization antiparallel to the lateral Bi displacements in the fluorite‐like layers. In compounds with even *n*, the antipolar order leads to complete cancelation of the out‐of‐plane components, confining the polarization strictly to the in‐plane *a*‐axis. In contrast, odd‐*n* compounds still exhibit dominant in‐plane polarization but retain a small additional out‐of‐plane component due to incomplete cancelation across the odd number of layers.^[^ ^50^ ^]^

Traditionally, the spacer layers in Aurivillius compounds are described as fluorite‐like [Bi_2_O_2_]^2+^ layers, characterized by a more covalent bonding nature. However, by analogy with *A*O and *B*O_2_ surface terminations in perovskite oxides,^[^ ^51^ ^]^ the two Bi layers within the fluorite‐like plane can alternatively be interpreted as belonging to vertically distorted BiO (*A*O) planes of a perovskite structure, separated by an intermediate 2O^4−^ atomic plane. This negatively charged oxygen double layer (highlighted in yellow in Figure 2) can significantly influence the electrostatic boundary conditions at interfaces between Aurivillius phases and other perovskite compounds.^[^ ^52^, ^53^, ^54^ ^]^

### 2.2 Carpy–Galy *A*_*n*_ *B*_*n*_O_3__*n*+2_ Compounds

The layered Carpy–Galy ferroelectrics comprise a variable number *n* of 110‐oriented perovskite planes interleaved with additional oxygen layers^[^ ^55^, ^56^, ^57^ ^]^ (Figure 2). The dominant ferroelectric instability in the Carpy–Galy phases originates from oxygen octahedral tilts/rotations,^[^ ^36^, ^58^ ^]^ which are commonly referred to as antiferrodistortive instabilities. In typical three‐dimensional perovskite crystals, such antiferrodistortive modes create local electric dipoles that cancel each other out, resulting in zero net polarization. However, in Carpy–Galy phases, the presence of oxygen spacer layers (highlighted in yellow) truncates the unit cell along the out‐of‐plane axis, creating an odd number of such electric dipoles between the spacers, which leads to a nonzero net in‐plane polarization (typically up to 10 μC cm^−2^ in bulk^[^ ^59^, ^60^ ^]^ and shown to reach 18 μC cm^−2^ in epitaxial thin films^[^ ^61^ ^]^). Because the polarization emerges from layering of the structure that creates this “dipole imbalance”, the phenomenon is referred to as geometric or topological ferroelectricity.^[^ ^58^ ^]^

The proper ferroelectricity in these compounds is characterized by oxygen octahedral tilts/rotations, *A*‐site lateral displacements, and slight *B*‐cation offcentering, schematized by red, green, and blue arrows in Figure 2, respectively. It is coupled to additional nonpolar distortions, such as additional octahedral tilting or antipolar distortions.^[^ ^61^ ^]^ Together, these distortions lower the symmetry to polar space groups such as *P*2_1_ or *Cmc*2_1_ with a unique in‐plane polarization axis. The multitude of polar and nonpolar distortions present in Carpy–Galy compounds suggests numerous plausible phase‐transition pathways between the high‐temperature symmetric and ferroelectric space groups,^[^ ^36^, ^61^ ^]^ which are challenging to disentangle, especially given that the *T*_c_ of these compounds is among the highest reported.^[^ ^62^ ^]^ This also gives rise to flat phonon bands, which are linked to experimentally observed incommensurate intermediate phases.^[^ ^63^, ^64^, ^65^, ^66^ ^]^

The highest polarization is typically observed for *n* = 4 Carpy–Galy compounds with the empirical formula *A*_2_ *B*_2_O_7_ (nominally identical to that of pyrochlore *A*_2_ *B*_2_O_7_,^[^ ^67^ ^]^ although their respective structural stabilities are governed by the size of the constituent cations^[^ ^68^ ^]^). By analogy with *A*O and *B*O_2_ surface terminations in perovskite oxides,^[^ ^51^ ^]^ the Carpy–Galy unit cell can be decomposed into *AB*O, O_2_, *B*O, and distorted *A*O_2_ atomic planes, where the additional oxygen spacer (highlighted in yellow) can be represented as two adjacent *A*O_2_ layers; see Figure 2.

### 2.3 Ruddlesden–Popper *A*_*n*+1_ *B*_*n*_O_3*n*+1_ Compounds

Ruddlesden–Popper phases comprise *n* perovskite layers separated by a double‐layer rock‐salt spacer (appears as two consecutive *A*O planes, see Figure 2). In their high‐symmetry parent structure, no unstable polar phonon exists; instead, two nonpolar zone‐boundary instabilities dominate: an in‐phase octahedral rotation about the out‐of‐plane axis and an antiphase tilt about an in‐plane axis.^[^ ^40^ ^]^ These two nonpolar modes enable the appearance of a polar mode as a result of a trilinear coupling.^[^ ^40^, ^69^, ^70^ ^]^ In this case, polarization is a secondary order parameter resulting from the product of the two primary rotations and tilts, in the so‐called avalanche phase transitions, where all three modes condense together. The resulting polar displacement pattern in Ruddlesden–Popper ferroelectrics consists of lateral displacements of the *A*‐site cations in each *A*O plane, which is represented by the green arrows in Figure 2. The direction of each displacement alternates from layer to layer. For even *n* the unit cell contains an odd number of *A*O planes within spacers, leading to a net in‐plane polarization. Conversely, for odd *n*, there is a cancelation between dipoles yielding a nonpolar ground state.

Because polarization is not the primary order parameter, switching it requires reversing the octahedral rotations; consequently, the coercive fields are higher and the spontaneous polarization is generally lower than in proper ferroelectrics (up to 4 μC cm^−2^^[^ ^70^ ^]^). There is a linear dependance between ferroelectric *T*_c_ and Goldschmidt tolerance factor in these phases, confirming the purely geometric polarization origin^[^ ^71^ ^]^ and enabling rational design^[^ ^72^, ^73^ ^]^ polarization in *A*‐site substituted compounds (*A*, *A’*)*B*_2_O_6_. On the one hand, to reduce the coercive field, the average tolerance factor of two perovskite layers (with *A* and *A*′ cations) should be maximized.^[^ ^73^ ^]^ On the other hand, the higher the difference in the tolerance factor between the two perovskite layers, the higher the polarization.^[^ ^73^ ^]^

Recently, Markov et al. identified a new structural family of anti‐Ruddlesden–Popper phases, with reversed cation and anion locations, through the analysis of a high‐throughput database of phonon band structures.^[^ ^74^ ^]^ In these compounds, the polarization is predicted to lie perpendicular to the layer stacking direction.

### 2.4 Dion–Jacobson *A*′[*A*_*n*‐1_ *B*_*n*_O_3*n*+1_] Compounds

The Dion–Jacobson phases consist of *n* perovskite slabs separated by a large single *A*′ cation layer instead of a double *A*O layer as in the Ruddlesden–Popper phases. *A*′ is typically a univalent alkali cation. Despite this structural difference, the mechanism of ferroelectricity in Dion–Jacobson compounds is remarkably similar to that of Ruddlesden–Popper phases: the polarization arises from a trilinear coupling between two nonpolar octahedral rotation/tilt modes.^[^ ^44^ ^]^ Similar to Ruddlesden–Popper ferroelectrics, those of the Dion–Jacobson family have moderate polarization (2–3 μC cm^−2^^[^ ^75^ ^]^) and high switching barriers. Due to its geometric origins, polarization can be induced through cation exchange.^[^ ^76^ ^]^

The hybrid–improper distortion drives lateral *A*‐site shifts (green arrows in Figure 2) and off‐center displacements of the *B* cations within the *B*O_6_ octahedra (blue arrows).^[^ ^43^ ^]^ First‐principles study shows that in *n* = 2 compounds, the *A*O layers supply a large share of the polarization, but the main contribution arises from the *B*O_2_ layers (as there are more of them).^[^ ^44^ ^]^

### 2.5 Common Ferroelectric Signatures: High *T*_C_ and In‐Plane Polarization

Ferroelectricity in these four phases is primarily stabilized by the layering and confinement effects of their large periodic unit cells rather than by orbital hybridization^[^ ^77^ ^]^ within the *AB*O_3_ units seen in conventional perovskite ferroelectrics like BaTiO_3_. Polarization stability determined on the larger scale means it often has superior stability and robustness compared to traditional ferroelectric perovskites. This is reflected in unusually high ferroelectric *T*_C_ values: around 600–800 °C in Aurivillius compounds,^[^ ^46^, ^78^ ^]^ above 1000 °C in Dion–Jacobson compounds,^[^ ^43^, ^79^ ^]^ and as high as 800 °C in Ruddlesden–Popper ferroelectrics,^[^ ^80^ ^]^ while with ca. 1500 °C, Carpy–Galy compounds boast the likely highest *T*_C_ of any known ferroelectric.^[^ ^62^ ^]^

All four families of layered perovskite ferroelectrics share a common structural motif: blocks of perovskite slabs separated by additional spacer layers. During crystal or thin‐film growth, these spacers usually align parallel to the substrate (or to the crystal facet with the largest surface area), since this orientation minimizes the surface energy.^[^ ^81^, ^82^ ^]^ Because the spacers carry a higher local ionic charge than the perovskite blocks, the structural anisotropy directly translates into anisotropy of the electrostatic boundary conditions. The charged layers preferentially screen the bound charges of the ferroelectric slabs, leading to cancelation of the out‐of‐plane polarization component (antipolar‐like order). In contrast, the in‐plane component remains uncompensated. Despite family‐specific microscopic mechanisms, the net macroscopic polarization in all four layered systems is therefore typically uniaxial and lies in‐plane, parallel to the spacer planes.

### 2.6 Opportunities and Challenges for Integrating In‐Plane‐Polarized Layered Ferroelectrics into Devices

The in‐plane polarization of naturally layered perovskite‐based ferroelectrics presents both a fundamental feature and a practical challenge for device integration. In particular, it requires interdigitated top electrodes with very small lateral separations: narrow gaps are necessary to generate the electric fields required to switch the polarization direction with relatively low voltages. With standard lithography techniques (e.g., projection lithography or electron‐beam lithography), one is limited to gaps of ≈1 μm between interdigitated electrodes in order to fully transfer the features in the resist and achieve a complete lift‐off.^[^ ^83^ ^]^ This is the reason why the fabrication of devices with in‐plane ferroelectrics has long been deemed impractical.

Early research attempted to circumvent this issue by forcing the layered unit cells to tilt with respect to the substrate surface normal, thereby reorienting the polarization to have a partial out‐of‐plane component.^[^ ^84^, ^85^, ^86^ ^]^ In parallel, first‐principles studies searched for Ruddlesden–Popper phases with spontaneous polarization along the out‐of‐plane axis, identifying a few promising non‐oxide candidates.^[^ ^87^ ^]^

More recently, however, several developments have revived interest in exploiting the intrinsic in‐plane polarization of these materials. Advances in extreme ultraviolet lithography (EUV) in the future will allow fabrication of nanoscale electrode features, enabling lateral gaps compatible with practical switching voltages and offering promising paths for future device architectures. In addition, all‐optical methods for ferroelectric‐polarization switching and readout have been demonstrated.^[^ ^88^, ^89^ ^]^ It was also shown that in some ferroelectrics, nanoscale metallic regions can be induced by electron‐beam‐triggered phase transitions,^[^ ^90^, ^91^ ^]^ which could enable direct writing of metallic contacts in the same material matrix. These emerging strategies suggest that the integration of in‐plane‐polarized layered ferroelectrics into devices could one day become technologically feasible.

## 3 Evolution of Thin‐Film Synthesis of Layered Ferroelectrics

The layered ferroelectric families were all discovered as bulk single crystals, where functional ferroelectric domains and polarization were probed for the first time. Device integration, however, demands thin films that are uniform over large areas, tolerant to heterogeneous stacking, and can be achieved through controlled synthesis (in terms of the correct phase, its orientation, and required thickness). The fabrication of layered ferroelectrics as thin films started as early as the late 1960s, and since then, the field has seen changes in synthesis techniques, choice of substrates, and characterization methods, which together have led to dramatic improvements in the thin‐film quality and associated functional properties.

### 3.1 Technique Evolution: From Ceramic Films to Modern Epitaxy

**Figure 3:**
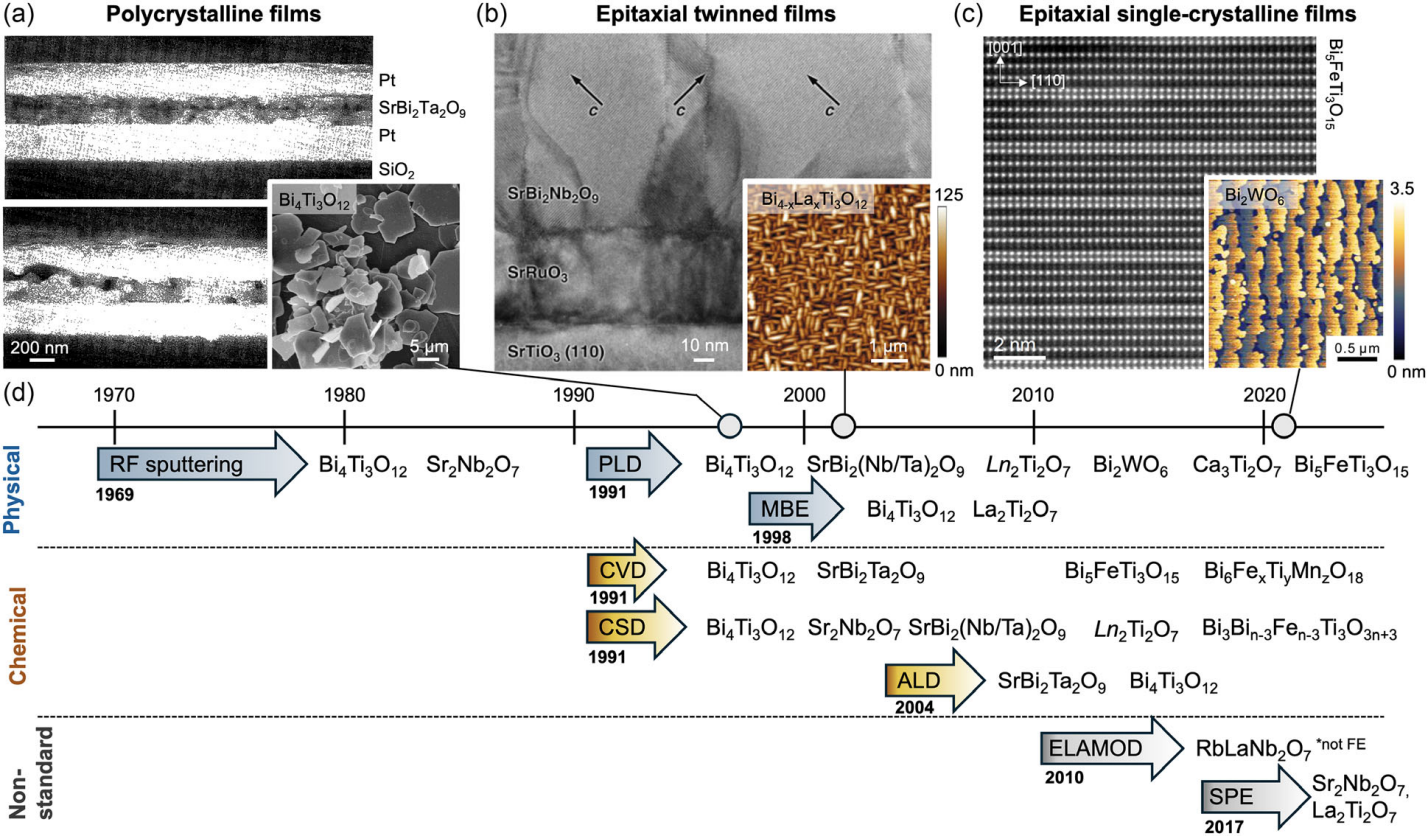
Historical evolution of deposition techniques and improvement in layered ferroelectric thin‐film quality. Representative layered ferroelectric oxides synthesized as thin films are shown across three stages of development: a) Polycrystalline films obtained by CSD methods, as seen in cross‐sectional scanning electron microscopy (SEM) micrographs of a typical SrBi_2_Ta_2_O_9_ capacitor stack before and after high‐temperature annealing.^[^ ^98^ ^]^ Reproduced with permission.^[^ ^98^ ^]^ Copyright 1996, IOP Publishing. SEM image (inset) revealing large Bi_4_Ti_3_O_12_ crystallites prepared using molten‐salt synthesis.^[^ ^99^ ^]^ Reproduced with permission.^[^ ^99^ ^]^ Copyright 1999, Wiley‐VCH GmbH. b) Epitaxial twinned films grown by PLD, showing tilted crystallographic domains in cross‐sectional transmission electron microscopy (TEM) of SrBi_2_Nb_2_O_9_.^[^ ^103^ ^]^ Reproduced with permission.^[^ ^103^ ^]^ Copyright 2000, AIP Publishing. La‐substituted SrBi_2_Ta_2_O_9_ topography with anisotropic grain structures.^[^ ^84^ ^]^ Adapted with permission.^[^ ^84^ ^]^ Copyright 2002, The American Association for the Advancement of Science. c) Atomically resolved layering in Aurivillius Bi_5_FeTi_3_O_15_ *n *= 4 films.^[^ ^104^ ^]^ Adapted with permission.^[^ ^104^ ^]^ Copyright 2021, American Chemical Society. Atomically flat terraces in Bi_2_WO_6_ films,^[^ ^209^ ^]^ demonstrating quality comparable to epitaxial perovskite oxide films. Adapted with permission.^[^ ^209^ ^]^ Copyright 2021, American Chemical Society. d) The timeline outlines the emergence of key thin‐film‐deposition methods grouped into physical, chemical, and nonstandard approaches and lists representative compositions successfully stabilized by each; see Table 1 for the study references.

The first report of layered ferroelectrics synthesized as thin films dates back to 1969, when Takei et al.^[^ ^92^ ^]^ used RF‐sputtering to produce Aurivillius Bi_4_Ti_3_O_12_ thin films on Pt and MgO. In the 1970s, sputtering was also utilized to obtain Carpy–Galy Sr_2_Nb_2_O_7_ films.^[^ ^93^ ^]^ It was not until 1990s that three independent advances arrived almost simultaneously. Ramesh et al. first used pulsed‐laser deposition (PLD) to grow Aurivillius Bi_4_Ti_3_O_12_ directly on epitaxial cuprate electrodes, demonstrating the feasibility of epitaxy on silicon wafers via an oxide buffer.^[^ ^94^, ^95^ ^]^ In the same year, Joshi et al. introduced a sol–gel process, one of the chemical‐solution deposition (CSD) approaches, to produce Bi_4_Ti_3_O_12_.^[^ ^96^ ^]^ Finally, Wills et al. used organometallic chemical vapor deposition (CVD) to deliver Aurivillius films.^[^ ^97^ ^]^ At that time, films were typically a few hundred nanometers thick and consisted of large, misoriented grains. Postannealing treatments were therefore required, but these often introduced additional surface roughness^[^ ^98^, ^99^ ^]^ (**Figure** **3**a). The fatigue‐free behavior in Aurivillius SrBi_2_Ta_2_O_9_ capacitors demonstrated in 1995^[^ ^30^ ^]^ fueled even more research efforts in the field. Molecular‐beam epitaxy (MBE) was first used in 1998, improving upon phase purity in Aurivillius films.^[^ ^100^ ^]^ Atomic layer deposition (ALD) soon followed: plasma‐enhanced ALD of SrBi_2_Ta_2_O_9_ enabled the thin‐film deposition with higher conformality and at lower temperatures.^[^ ^101^, ^102^ ^]^ Figure 3d shows the chronological development of deposition methods used to prepare layered ferroelectric thin films.

A more detailed overview of reported layered perovskite‐based ferroelectric oxide thin films is provided in **Table** **1**, summarizing key materials across the Aurivillius, Carpy–Galy, Ruddlesden–Popper, and Dion–Jacobson families, which have been synthesized as thin films. Over the years, PLD has been the preferred physical vapor deposition method, with most reported layered ferroelectrics grown using it. Among chemical deposition methods, CVD and sol–gel have become standard synthesis approaches for these materials.

**Table 1:** Overview of reported layered ferroelectric perovskite‐based oxide thin films: crystal family (A = Aurivillius; CG = Carpy–Galy; RP = Ruddlesden–Popper; DJ = Dion–Jacobson), number *n* of perovskite layers, substrates, growth techniques, film quality, remanent polarization *P*, coercive field *E*_c_, domain imaging method, and associated references. Substrates: SrTiO_3_ (STO), LaAlO_3_ (LAO), DyScO_3_ (DSO), NdGaO_3_ (NGO), (LaAlO_3_)_0.3_(Sr_2_AlTaO_6_)_0.7_ (LSAT).

Material	Class	*n*	Substrate	Technique	Film quality	*P* [μC cm^−2^]	E_c_ [kV cm^−1^]	Domains	Ref.
SrBi_2_Nb_2_O_9_	A	2	STO (110), STO (001)	PLD, sol–gel	epitaxial, twinned	22.8	200	–	[103, 124, 212]
SrBi_2_Ta_2_O_9_	A	2	Pt/TiO_*x*_/SiO_2_/Si, graphite	CSD, MOCVD, ALD, PLD	polycrystalline	–	–	–	[30, 98, 101, 213, 214, 215, 216]
SrBi_2_Ta_2_O_9_	A	2	LAO (001), STO (001)	PLD	epitaxial, twinned	–	–	–	[212]
Sr_5_Bi_4_Ti_8_O_27_	A	8	STO (001)	PLD	epitaxial	–	–	–	[217]
Bi_4_Ti_3_O_12_	A	3	quartz; Pt/SiO_2_/Si; sapphire	sol–gel, MOCVD, ALD	polycrystalline	0.5 (ALD)	–	–	[96, 97, 102, 218, 219]
Bi_4_Ti_3_O_12_	A	3	Pt; STO (001); LAO (001); MgAl_2_O_4_	RF–sputtering, PLD, MBE, CVD	epitaxial, twinned	–	–	PFM	[92, 94, 95, 100, 117]
La‐doped Bi_4_Ti_3_O_12_	A	3	SrRuO_3_/YSZ/Si(100)	PLD	epitaxial, twinned, minor misorientation	32	265	–	[84, 85]
Bi_2_WO_6_	A	1	STO (100), LAO (100)	PLD	epitaxial, twinned	–	–	PFM, STM	[86, 127, 155, 209, 220, 221]
Bi_5_FeTi_3_O_15_	A	4	Pt/Si; Si(100); NGO (001); STO (100)	CSD, PLD, AVD	polycrystalline, oriented	20.8, 16.5	250	PFM, STM	[53, 54, 104, 105, 143, 179, 222, 223]
Bi_6_Fe_2_Ti_3_O_18_	A	5	Pt/Si; sapphire	CSD, AVD	polycrystalline	24	300	–	[179, 180]
Bi_7_Fe_3_Ti_3_O_21_	A	6	Pt/Si; NGO (001)	CSD, PLD	polycrystalline	22.4	300	PFM, STM	[104, 179]
Bi_9_Fe_5_Ti_3_O_27_	A	8	NGO (001)	PLD	polycrystalline	–	–	PFM, STM	[104, 179]
Bi_6_Ti_x_Fe_y_Mn_z_O_18_	A	5	NGO (001), LSAT (100), STO (100)	DLI‐CVD	epitaxial/polycrystalline	–	–	PFM	[144, 151, 152, 224]
BaBi_4_Ti_4_O_15_	A	4	Nb:STO (001)	PLD	epitaxial, twinned	–	–	PFM	[225]
La_2_Ti_2_O_7_	CG	4	STO (110), STO (100), LSAT (110), DSO (100)	MBE, PLD, sol–gel	epitaxial single‐crystal/twinned	18.3	16.9	PFM, STEM	[61, 68, 110, 112, 126, 129, 226, 227, 228, 229, 230]
Sr_2_Nb_2_O_7_	CG	4	Sr_2_Ta_2_O_7_ (single crystal)	RF–sputtering	oriented	–	–	–	[93]
Sr_2_Nb_2_O_7_	CG	4	Si(100), Pt‐coated Si(100)	sol–gel	polycrystalline	–	–	–	[231]
Sr_2_Nb_2_O_7_	CG	4	STO (110), LAO (110)	PLD, solid‐phase epitaxy	epitaxial	–	–	–	[90, 113, 232]
Nd_2_Ti_2_O_7_	CG	4	STO (110)	PLD, sol–gel	epitaxial	–	–	–	[68, 111, 156, 230]
Pr_2_Ti_2_O_7_	CG	4	STO (110)	PLD, sol–gel	epitaxial	–	–	–	[68, 230]
Ce_2_Ti_2_O_7_	CG	4	STO (110)	PLD, sol–gel	epitaxial	–	–	–	[68, 230]
Sm_2_Ti_2_O_7_	CG	4	STO (110)	PLD, sol–gel	epitaxial	–	–	–	[68, 230]
Ca_3_Ti_2_O_7_	RP	2	STO (110)	PLD	epitaxial, twinned	8 (at 2 K)	5 (at 2 K)	–	[233]
Ca_3_Mn_2_O_7_	RP	2	STO (100)	PLD	polycrystalline	–	–	–	[234]
CsBiNb_2_O_7_	DJ	2	LAO (001); free‐standing	molten‐salt‐assisted synthesis	epitaxial	–	–	STEM	[134, 235]

### 3.2 Improvement in Thin‐Film Textures Through Epitaxy

The 2000s saw improvements in thin‐film textures as researchers transitioned from growing layered ferroelectrics on Pt/Si/glass wafers to using single‐crystalline perovskite oxide substrates. This led to epitaxially oriented films, which enabled controlling the majority‐grain orientation and therefore increased net switchable polarization in the films.^[^ ^84^, ^103^ ^]^ However, since anisotropic unit cells of layered ferroelectrics cannot be fully epitaxially matched by cubic perovskite substrates (Section 4.2), the films still suffered from crystallographic twins and rather grainy structures, see Figure 3b. True single‐crystallinity in epitaxial films emerged only when both lattice‐ and symmetry‐matched substrates became available and were chosen for synthesis. For instance, Aurivillius Bi_*n*+1_Fe_*n*−3_Ti_3_O_3*n*+3_ films were grown by PLD on NdGaO_3_ substrates with the orthorhombic (001) orientation (the more common NdGaO_3_ cut is pseudocubic (110)). This approach yielded twin‐free, single‐crystalline films with uniaxial in‐plane polarization and atomically flat surfaces^[^ ^104^, ^105^ ^]^ (Figure 3c). Aside from improving thin‐film quality and reducing crystallographic twinning, epitaxial strain has also been predicted to serve as an additional tuning knob for polarization in layered ferroelectrics. Theoretically, it has been shown to drive phase transitions between polar, nonpolar, and antiferroelectric states.^[^ ^106^, ^107^, ^108^, ^109^ ^]^

### 3.3 Nonconventional Synthesis Methods Driven by Structural Anisotropy

While the anisotropic stacking of layered perovskites is often viewed as a crystal‐growth challenge, that same anisotropy can be leveraged for unconventional growth strategies. One such approach is solid‐phase epitaxy (SPE), where an amorphous or metastable precursor film is deposited at low temperature and subsequently annealed. This method has proven effective for stabilizing kinetically demanding Carpy–Galy layered phases (e.g., Sr_2_Nb_2_O_7_ and La_2_Ti_2_O_7_).^[^ ^110^, ^111^ ^]^ When annealed in an oxygen atmosphere, these materials crystallize with their spacer layers preferentially aligned parallel to the substrate.^[^ ^61^, ^112^, ^113^ ^]^ This behavior contrasts with isotropic cubic perovskites, where recrystallization typically leads to randomly oriented grains and twinning. In layered ferroelectrics, by contrast, the inherent structural anisotropy acts as a self‐templating guide, enabling the recovery of epitaxial alignment, hence the term “solid‐phase epitaxy”. Nevertheless, multisite nucleation during annealing can still result in stacking faults, grain boundaries, and surface roughness.

This limitation can be addressed by an alternative strategy known as excimer‐laser‐assisted metal‐organic deposition (ELAMOD), developed by Nakajima and coworkers.^[^ ^114^, ^115^, ^116^ ^]^ ELAMOD similarly capitalizes on the structural anisotropy of layered materials, but initiates crystallization through localized energy input. A pulsed excimer laser selectively heats only the film surface, triggering crystallization exclusively from the top surface. The resulting growth front then gradually propagates through the film thickness, maintaining a uniform crystal orientation and significantly reducing the likelihood of stacking defects. Although demonstrated on non‐ferroelectric Dion–Jacobson compounds such as RbLaNb_2_O_7_ and RbCa_2_Nb_3_O_10_, the method shows how anisotropic structure can be exploited to achieve directional, self‐aligned film growth even at low temperature on non‐lattice matching substrates like glass.^[^ ^115^ ^]^

## 4 Structural and Functional Characterization of Layered Perovskite‐Based Ferroelectrics

The structural and functional characterization of layered perovskite‐based ferroelectrics builds on techniques established for conventional perovskites, but their large unit cells introduce additional challenges. For instance, reflection high‐energy electron diffraction (RHEED) can track growth dynamics, yet the large periodicities of layered oxides often lead to multiple oscillations per unit cell that complicate interpretation (Section 4.1). In X‐ray diffraction (XRD), the large unit cells produce many additional reflections compared to simple perovskites, as well as artifacts related to stacking defects, increasing the complexity of the data (Section 4.2). In scanning transmission electron microscopy (STEM), mapping polarization becomes more involved because several distortion modes may contribute simultaneously, rather than just the simple *B*‐site off‐centering familiar from conventional ferroelectric perovskites (Section 4.3). Finally, in scanning probe microscopy, the strong structural anisotropy means that crystal orientation critically affects the measurement, with some geometries providing better access to polarization (Section 4.4). In this part, we review how these techniques are applied to layered perovskite‐based ferroelectrics, with a focus on the specific considerations and adaptations they require.

### 4.1 In Situ Diagnostics

The superlattice structure of layered perovskite‐based ferroelectrics forms spontaneously due to unit‐cell electrostatics, without the need for specific epitaxial strain or manual layering. This represents a clear advantage over artificial superlattices such as (PbTiO_3_/SrTiO_3_)_*n*_.^[^ ^23^, ^24^, ^25^ ^]^ As a result, the deposition of layered ferroelectric films can be achieved using a single precursor with the correct stoichiometry. Provided that the appropriate growth parameters are chosen to access the thermodynamic stability window of the desired phase, one can expect to stabilize the correct layered structure. For example, PLD growth uses a single ceramic target with the desired stoichiometry, making the synthesis efficient and straightforward. However, techniques such as MBE and CVD might still rely on an alternating sequence of precursors. To achieve Bi_4_Ti_3_O_12_ by MBE, Bi and Ti fluxes are introduced separately during growth,^[^ ^100^ ^]^ whereas CVD growth involves timed injections of Bi(thd)_3_ (thd = 2,2,6,6‐tetramethyl‐3,5‐heptanedionate) and Ti(*O*‐iPr)_2_(thd)_2_ (where *O*‐iPr = iso‐propoxide) solutions.^[^ ^117^ ^]^ For some growth methods that allow in situ diagnostics, the first characterization of layered ferroelectric thin films can already be carried out during deposition.

**Figure 4:**
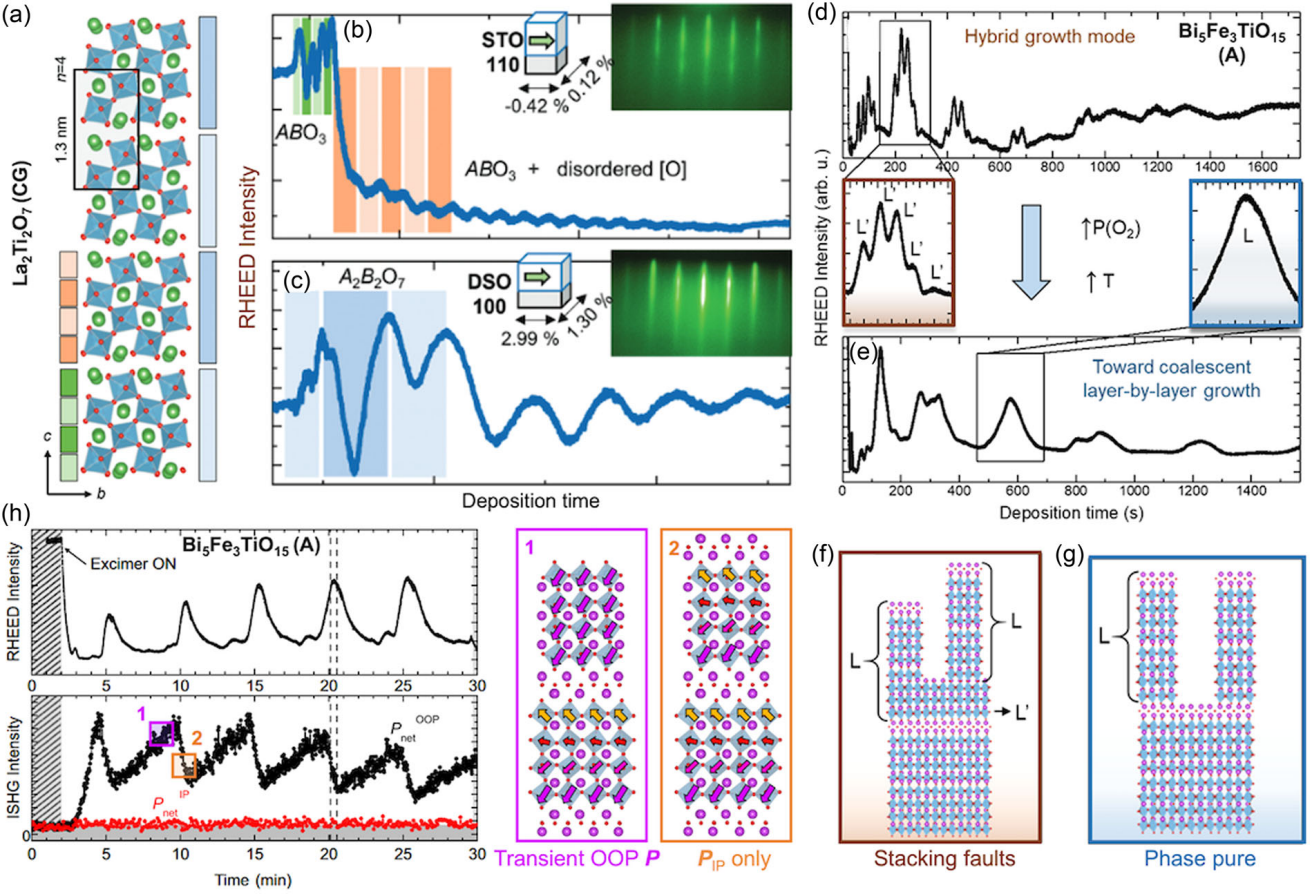
In situ monitoring of structure and polarization evolution during the growth of layered ferroelectrics. a–c) RHEED tracking of La_2_Ti_2_O_7_ (Carpy–Galy) film (a) growth:^[^ ^61^ ^]^ (b) growth in perovskite‐height layers on STO (110) leads to non‐ferroelectric polymorphs, and (c) well‐defined Carpy–Galy unit‐cell growth on DSO (100) leads to the desired phase. Adapted with permission.^[^ ^61^ ^]^ Copyright 2025, Wiley‐VCH GmbH. d–g) RHEED monitoring of Bi_5_FeTi_3_O_15_ (Aurivillius) growth:^[^ ^104^ ^]^ (d) hybrid mode and (e) coalescent layer‐by‐layer mode. Schematics (f,g) illustrate that only the latter suppresses stacking faults and yields a phase‐pure film. Adapted with permission.^[^ ^104^ ^]^ Copyright 2021, American Chemical Society. h) Simultaneous monitoring of Bi_5_FeTi_3_O_15_ growth with RHEED and ISHG signals.^[^ ^54^ ^]^ The oscillating ISHG signal reflects the transient emergence of out‐of‐plane polarization synchronized with structural layering (1,2). Adapted with permission.^[^ ^54^ ^]^ Copyright 2025, Springer Nature.

Reflection high‐energy electron diffraction (RHEED) is available for PLD, MBE, and sputtering, and it gives insights into how the crystallinity of the film develops during the growth and, in particular, on how the layering of perovskite planes and spacers is evolving. Ex situ X‐ray reflectivity and atomic force microscopy (AFM) can be used in combination with RHEED monitoring to disentangle the structural origin of RHEED oscillations.^[^ ^105^ ^]^ For instance, the growth of La_2_Ti_2_O_7_ Carpy–Galy *n* = 4 phase (**Figure** **4**a) on different substrates yields two distinct oscillation regimes.^[^ ^61^ ^]^ If the RHEED intensity trace shows rapid oscillations (Figure 4b), each corresponding to perovskite unit‐cell thickness, the film is growing as kinetically favored perovskite‐type or other nonpolar polymorphs. In order to achieve the CG phase in the as‐grown films, one should aim to stabilize large‐amplitude oscillations whose period matches the 1.3 nm height of an electroneutral *A*_2_ *B*_2_O_7_ block (Figure 4c). Such a growth mode can be referred to as a “coalescent” layer‐by‐layer mode unique to layered oxides. Instead of adding individual perovskite planes sequentially, electroneutral (half‐)unit‐cell blocks nucleate separately and laterally merge into complete atomically flat layers. First demonstrated for the *n* = 4 Aurivillius member using PLD,^[^ ^105^ ^]^ the approach was later extended to *n *= 6 and *n* = 8 films.^[^ ^104^ ^]^ Signatures of such growth mode have also been reported in *n* = 5 Aurivillius films grown by CVD.^[^ ^118^ ^]^

Once the correct layered phase is stabilized, RHEED intensity oscillations can help to identify different *n* intergrowths. For Bi_5_FeTi_3_O_15_ *n* = 4 films of the Aurivillius phase, “hybrid” growth modes can be observed, identified by the large unit‐cell oscillations modulated by smaller ones (Figure 4d). This signals that the perovskite planes are nucleating simultaneously with the large electroneutral layered unit cells. The result is an array of vertical stacking faults with higher *n* (Figure 4f). It was reported that raising the substrate temperature and oxygen pressure suppresses this mode (Figure 4e) and leads back into the coalescent growth mode that helps to achieve fault‐free films (Figure 4g).

**In situ second‐harmonic generation (ISHG)** offers a powerful, real‐time optical tool to monitor symmetry breaking and polar order during film growth.^[^ ^119^, ^120^ ^]^ A femtosecond laser is coupled into the growth chamber, and the second‐harmonic signal emitted from the thin film is collected. In centrosymmetric crystals, SHG generation is forbidden by symmetry. However, when inversion symmetry is broken by emergent polar order, SHG becomes allowed, and a finite signal appears. By selecting specific input/output light‐polarization configurations, the SHG response can be made sensitive to either in‐plane or out‐of‐plane (OOP) polarization components. The ISHG experiment tracking OOP polarization during the growth of the Aurivillius *n* = 4 thin film^[^ ^54^ ^]^ shows a sawtooth‐like modulation closely related to structural RHEED oscillations (Figure 4h). The SHG intensity gradually increases as each TiO_6_‐based perovskite block is deposited and abruptly drops when the block is capped by the negatively charged Bi_2_O_2_ spacer. This reveals that the transient OOP polarization arises from the electrostatic potential building up in the incompletely screened perovskite slab (Figure 4h box 1). When the Bi_2_O_2_ layer is deposited, it restores charge neutrality of the unit cell. As a result, the OOP component cancels out, and the unit cell reverts to the expected antipolar out‐of‐plane order, leaving only the in‐plane polarization component (Figure 4h box 2). This provides time‐resolved evidence that Bi_2_O_2_ layers govern the electrostatic landscape that stabilizes ferroelectricity in Aurivillius thin films.

### 4.2 XRD‐Based Techniques

XRD is typically the first method of choice for characterizing layered ferroelectric thin films, as it provides a full‐volume probe of the crystalline structure. Unlike surface‐sensitive techniques, XRD can detect parasitic phases or misoriented domains throughout the film thickness. Here, we highlight some XRD‐based approaches unique to layered ferroelectric thin films.

#### 4.2.1 Periodicity Faults Tracked by Peak Shape

**Figure 5:**
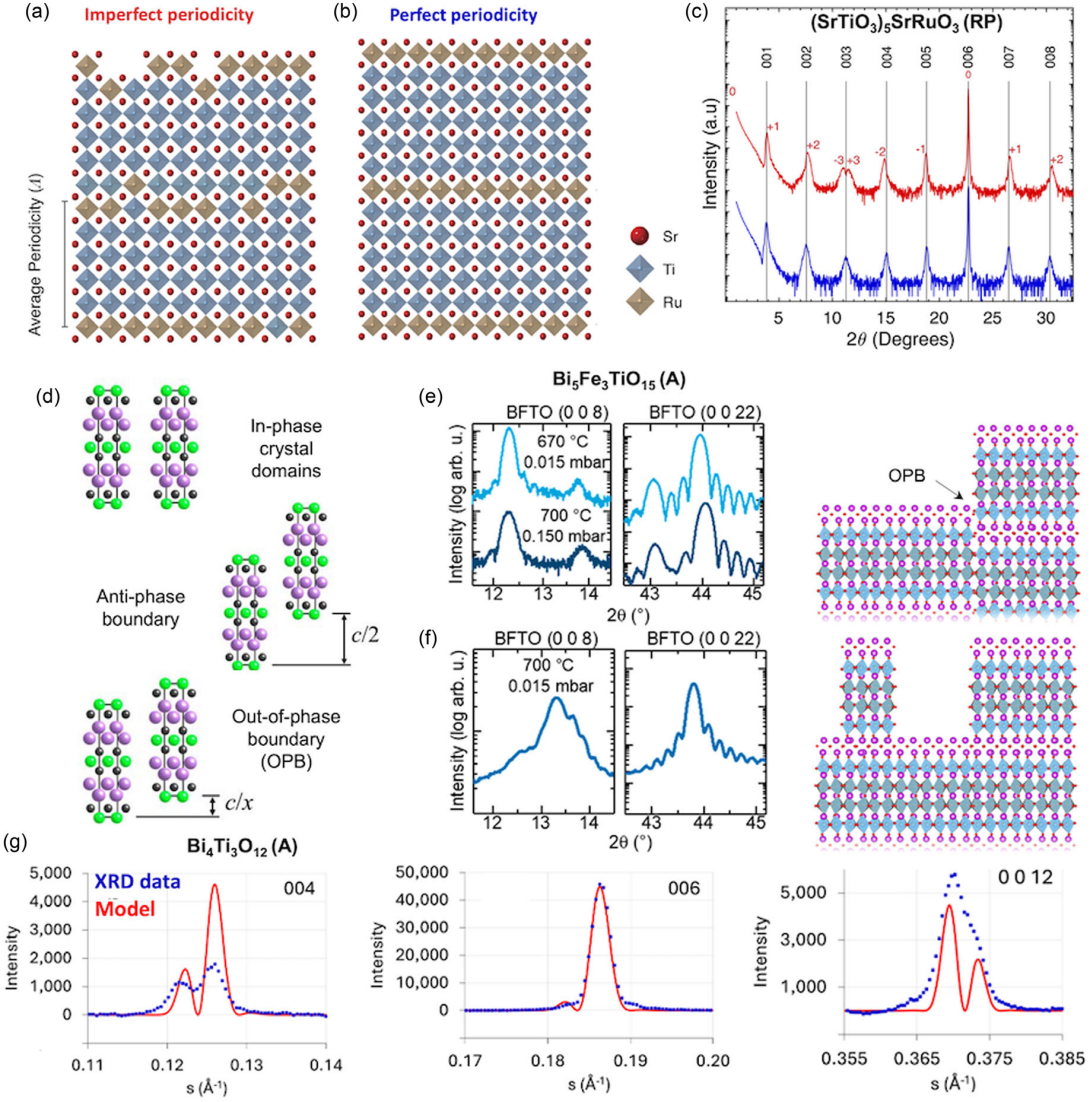
Diagnosing and modeling stacking faults in layered ferroelectric films by XRD. a–c) Ruddlesden–Popper superlattices ${\left({\text{SrTiO}}_{3}\right)}_{5}{\text{SrRuO}}_{3}$ grown by MBE:^[^ ^121^ ^]^ (a) ideal *n* = 5 periodicity and (b) imperfect stacking with average periodicity of 0.9 unit‐cell; (c) corresponding θ−2θ scans, where deviations from the ideal sequence (red) broaden or even split the reflections (here in superlattice notation) compared with the phase‐pure stack (blue). Reproduced with permission.^[^ ^121^ ^]^ Copyright 2021, AIP Publishing. d) Schematic illustrating in‐phase unit cells, an antiphase boundary where unit cells are shifted by exactly half a unit cell, and an out‐of‐phase boundary (OPB) where adjacent unit cells are misaligned by a fractional shift.^[^ ^210^ ^]^ Adapted with permission.^[^ ^210^ ^]^ Copyright 2005, Springer Nature. e,f) OPB control in Bi_5_FeTi_3_O_15_ (Aurivillius, n=4) films:^[^ ^104^ ^]^ (e) splitting of the (00ℓ) peaks indicates OPB defects, (f) whereas improved growth conditions eliminate the splitting and yield defect‐free films. Adapted with permission.^[^ ^104^ ^]^ Copyright 2021, American Chemical Society. g) Simulated XRD profiles from the OPB model (red) accurately reproduce the experimental Bi_4_Ti_3_O_12_ thin‐film data (blue) for the 0 0 4, 0 0 6, and 0 0 12 reflections.^[^ ^123^ ^]^ Reproduced with permission.^[^ ^123^ ^]^ Copyright 2025, International Union of Crystallography.

Layered ferroelectric films often display imperfect periodicity (**Figure** **5**a) despite sharing the same stoichiometry as perfectly periodic samples (Figure 5b), rendering many conventional characterization techniques insensitive to these structural variations. In *θ*–2*θ* scans, periodicity faults broaden, skew, or split the XRD peaks (Figure 5c). Barone et al.^[^ ^121^ ^]^ exploited this lineshape signature to iteratively tune flux ratios during MBE growth of Ruddlesden–Popper phases: successive runs sharpened the reflections and eventually enabled metastable (SrTiO_3_)_20_‐SrO *n* = 20 stacks with perfect periodicity.

#### 4.2.2 Identification and Modeling of Out‐Of‐Phase Boundaries

When adjacent unit cells are perfectly aligned along the stacking axis, no defect is present. An anti‐phase boundary forms when one unit cell is shifted vertically by exactly half a unit cell relative to its neighbor (Figure 5d). If the structural shift differs from half a unit cell, the result is an out‐of‐phase boundary (OPB). OPBs are the most widespread defect in layered perovskite oxides, and they most often nucleate at substrate steps, but can also arise from misfit dislocations or crystallographic shear when volatile species are lost during growth.^[^ ^122^ ^]^

While observed in all layered ferroelectrics, OPBs are mostly investigated in Aurivillius films, where they give rise to XRD‐peak splitting. For instance, in Bi_5_FeTi_3_O_15_, the OPBs split specific (00ℓ) peaks, e.g., 0 0 8 and 0 0 22^[^ ^104^ ^]^ (Figure 5e). Changing PLD growth conditions by raising temperature and oxygen partial pressure narrows or removes the peak split, indicating fewer OPBs (Figure 5f). Whatmore et al.^[^ ^123^ ^]^ introduced an analytical model that links physically accessible OPB parameters, such as the fractional unit‐cell shift across the OPB, the inclination angle at the film–substrate interface, and the average lateral spacing, to the resulting peak profile. With these inputs, the model accurately predicts which (00ℓ) reflections split and by how much, as confirmed by its agreement with the measured *θ*–2*θ* pattern of a PLD‐grown Bi_4_Ti_3_O_12_ film, see Figure 5g. Another approach to quantitatively evaluate the nature and degree of stacking faults is to model diffuse X‐ray scattering, as demonstrated for SrBi_2_Nb_2_O_9_ thin films.^[^ ^124^ ^]^ This allows for evaluation of the degree, scale, and evolution of planar defects in layered ferroelectric thin films.

Structural defects are often the link between the film microstructure and device performance in layered ferroelectrics. OPBs, although often labeled as “defects”, can play a beneficial role. In Aurivillius compounds, the celebrated fatigue‐free switching^[^ ^30^ ^]^ is attributed to the ability of the crystal to convert excess charged point defects into charge‐neutral OPBs:^[^ ^122^, ^125^ ^]^ they accommodate the compositional imbalance without degrading the ferroelectric response. In Carpy–Galy phases, OPBs that appear during growth act as oxidation pathways that allow diffusion of extra oxygen, thereby stabilizing the layered structure starting from the perovskite phase.^[^ ^126^ ^]^ Thus, rather than being purely detrimental, OPBs can serve as self‐compensating or self‐stabilizing features that aid the functional behavior of layered ferroelectric thin films.

#### 4.2.3 Crystallographic Twinning from Substrate Mismatch

Unlike conventional perovskite ferroelectrics such as PbTiO_3_ or BaTiO_3_, whose pseudocubic symmetry allows straightforward cube‐on‐cube epitaxy on common substrates like SrTiO_3_, epitaxy of layered ferroelectrics poses additional challenges. Their unit cells are typically orthorhombic or even monoclinic, with two different in‐plane lattice parameters and a polar axis that lies within the plane of the film. As a result, direct growth on cubic substrates often leads to crystallographic twinning, where the film adopts multiple in‐plane orientations to accommodate the symmetry mismatch. This not only affects film crystallinity but also suppresses net macroscopic polarization due to the presence of 90° ferroelastic domain variants.

**Figure 6:**
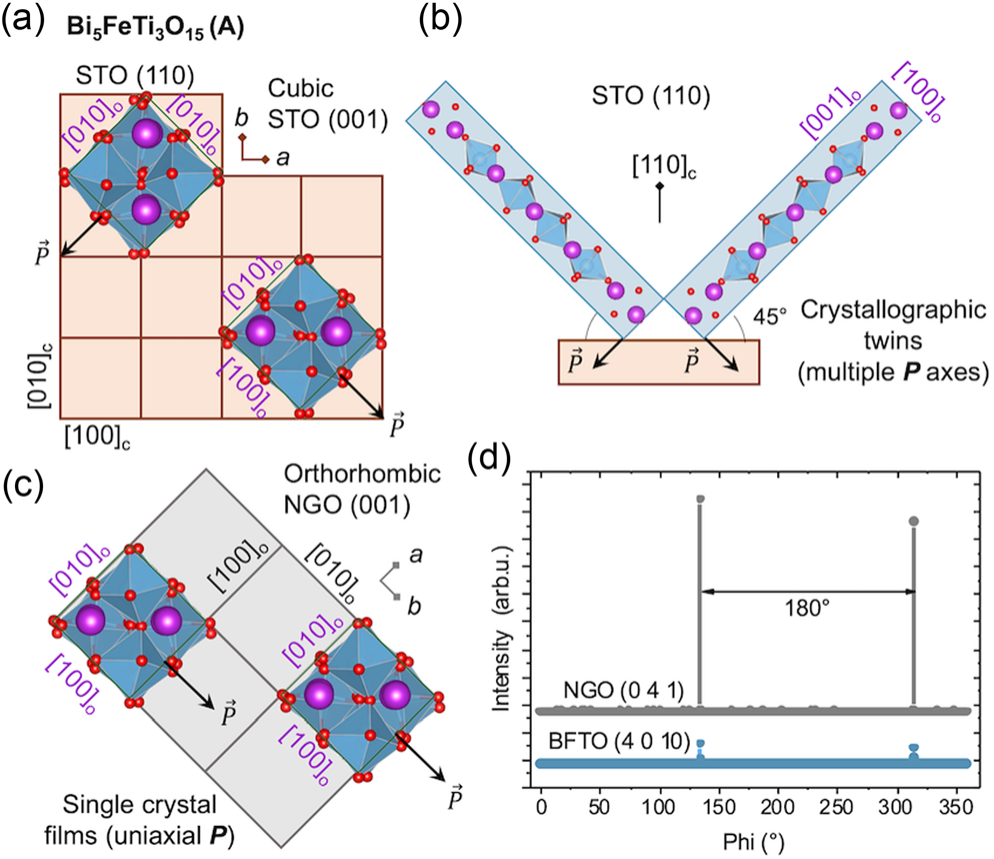
Epitaxial matching of orthorhombic Aurivillius layered ferroelectrics. a) In‐plane view of an orthorhombic Bi_5_FeTi_3_O_15_ (BFTO) unit cell on a cubic SrTiO_3_ (001) substrate. Lattice‐vector mismatch enforces four in‐plane polarization variants and generates 90° ferroelectric‐polarization twins (can be regarded as ferroelastic domains^[^ ^127^ ^]^). b) When BFTO is grown on SrTiO_3_ (110): the polar axis tilts 45° from the surface normal, again producing twin domains and a finite out‐of‐plane polarization component. c) On an orthorhombic, lattice‐matched NdGaO_3_ (001) substrate, the BFTO *a*‐ and *b*‐axes register one‐to‐one with the substrate, giving rise to a single‐crystal film with no twinning and a uniaxial in‐plane polarization. d) XRD *ϕ*‐scans confirm single‐crystal films: the BFTO (4010) reflection (blue) coincides with the twofold NdGaO_3_ (041) peaks (gray), demonstrating complete epitaxial matching.^[^ ^211^ ^]^ Adapted with permission.^[^ ^211^ ^]^ Copyright 2022, ETH Zurich.

**Figure** **6** illustrates this issue using the Aurivillius Bi_5_FeTi_3_O_15_ (BFTO) films as an example. When grown on SrTiO_3_ (001) (Figure 6a), Aurivillius films form four rotational crystallographic and polarization variants due to in‐plane symmetry mismatch.^[^ ^127^ ^]^ On SrTiO_3_ (110) (Figure 6b), crystallographic twins are also present with the polar axis tilting out of the plane, giving rise to an out‐of‐plane polarization component.^[^ ^86^, ^103^ ^]^ However, when BFTO is grown on orthorhombic NdGaO_3_ (001), whose symmetry and lattice constants match those of BFTO^[^ ^104^, ^105^, ^118^ ^]^ (Figure 6c), the film adopts a single‐crystal structure with matched *a*‐ and *b*‐axes and, therefore, a uniaxial in‐plane polarization along a single substrate axis. This can be confirmed by X‐ray *ϕ*‐scans (Figure 6c), where the BFTO (4 0 10) peaks match the twofold symmetry of the NdGaO_3_ (0 4 1) reflection.^[^ ^105^ ^]^ This example emphasizes that layered ferroelectrics require careful substrate selection to achieve both lattice and unit‐cell symmetry matching.

### 4.3 STEM

STEM has become a key technique for imaging layered ferroelectric oxides at the atomic scale. It enables direct visualization of unit‐cell layering, local polar displacements, mapping of ferroelectric domains, and the presence of structural defects such as OPBs or stacking faults.

#### 4.3.1 Mapping of *B*‐Site Off‐Centering in Aurivillius Compounds

**Figure 7:**
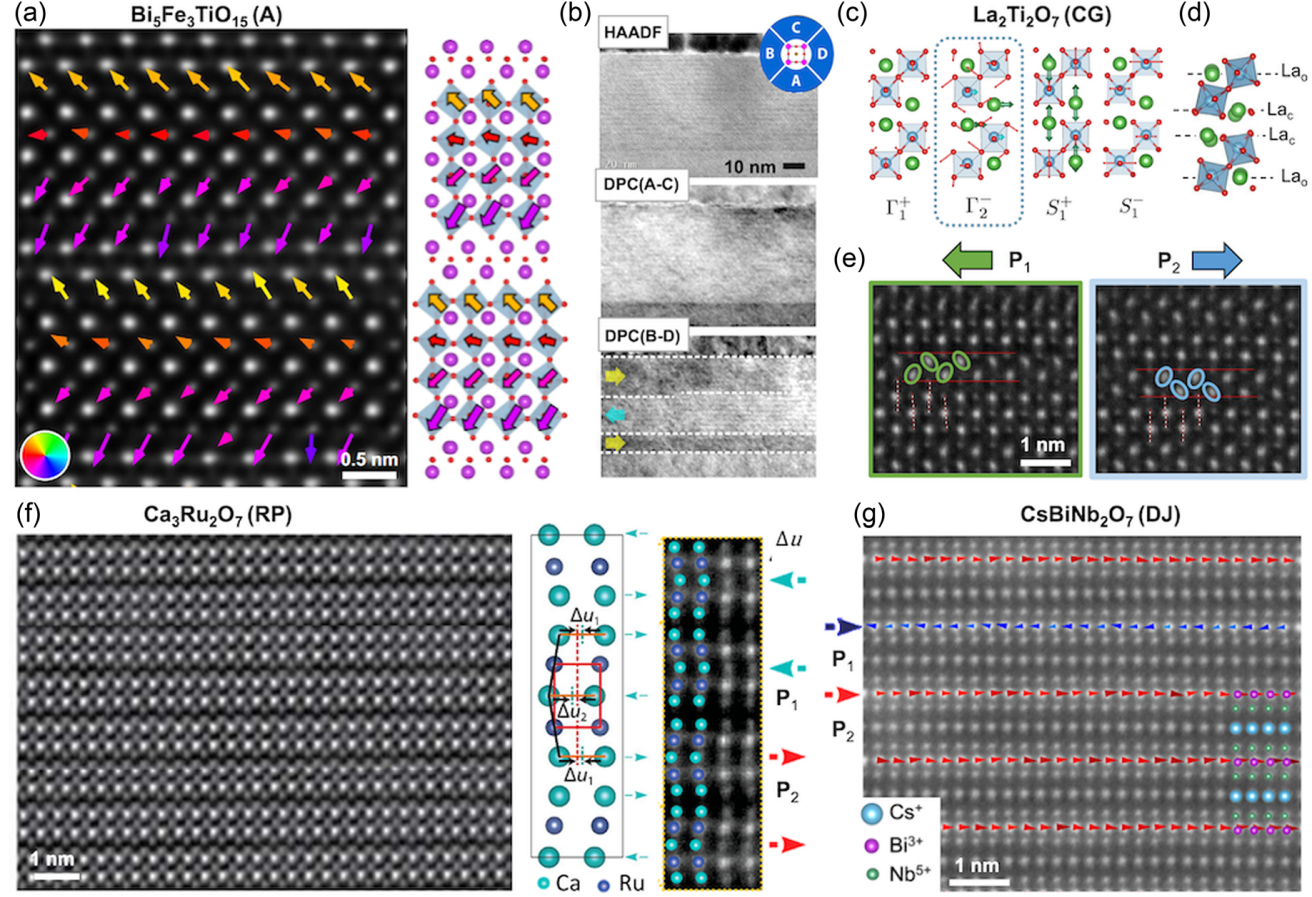
Atomic‐scale mapping of polar displacements in layered ferroelectrics by STEM. (a,b) Bi_5_FeTi_3_O_15_ (Aurivillius): (a) HAADF‐STEM overlaid with dipole vectors reveals antipolar vertical shifts of TiO_6_ octahedra toward neighboring Bi_2_O_2_ layers with a net in‐plane polarization.^[^ ^54^ ^]^ Adapted with permission.^[^ ^54^ ^]^ Copyright 2025, Springer Nature. (b) Large‐area HAADF and DPC images show out‐of‐plane (A–C) and in‐plane (B–D) electric‐field components. The latter gives access to large‐scale imaging of buried in‐plane domains.^[^ ^128^ ^]^ Reproduced with permission.^[^ ^128^ ^]^ Copyright 2019, American Chemical Society. c–e) La_2_Ti_2_O_7_ (Carpy–Galy): symmetry‐mode analysis, (c) links distortion ($\Gamma_{2}^{-}$) to the ferroelectric $P2_{1}$ unit cell, (d) HAADF‐STEM, and (e) shows that the lateral direction of La “teardrop’’ doublets (La_c_ in (d)) are indicative of in‐plane polarization direction.^[^ ^61^ ^]^ Adapted with permission.^[^ ^61^ ^]^ Copyright 2025, Wiley‐VCH GmbH. (f) Ca_3_Ru_2_O_7_ (Ruddlesden–Popper): ADF‐STEM image reveals bilayer structure with *A*‐site lateral off‐center displacements, with the direction alternating from layer to layer. The net *A*‐site off‐center displacement within each bilayer is $\Delta u=2\Delta u_{1}-\Delta u_{2}$ and is used to map in‐plane polarization directions.^[^ ^130^ ^]^ Adapted with permission.^[^ ^130^ ^]^ Copyright 2018, American Chemical Society. (g) CsBiNb_2_O_7_ (Dion–Jacobson): HAADF‐STEM with Bi^3+^ displacements relative to Cs^+^ columns are used to map polarization, revealing unit‐cell‐wide 180° ferroelectric domains.^[^ ^134^ ^]^ Reproduced with permission.^[^ ^134^ ^]^ Copyright 2021, American Physical Society.

In Aurivillius phases, the polar mode can be described by the simultaneous and antiparallel shifts of the Bi_2_O_2_ layers and the perovskite oxygen octahedra. While both the lateral displacement of Bi cations and the off‐centering of *B*‐site cations within oxygen octahedra can be imaged,^[^ ^128^ ^]^ most studies focus on mapping the *B*‐site cation displacements. This approach is sufficient to determine the polarization direction and follows established procedures from conventional perovskite ferroelectrics. As shown in **Figure** **7**a, high‐angle annular dark‐field (HAADF‐) STEM imaging reveals antipolar stacking of *B*‐site cation displacements along the out‐of‐plane direction and a net in‐plane polarization within each perovskite block.^[^ ^53^, ^54^, ^129^ ^]^ Additionally, differential phase contrast (DPC‐) STEM has been proposed as an efficient technique for rapid, large‐area mapping of buried in‐plane domains in Aurivillius compounds^[^ ^128^ ^]^ (Figure 7b). This technique relies on a four‐quadrant segmented annular dark‐field detector to resolve the projected electric‐field distribution.

#### 4.3.2 Octahedral Rotation‐Driven Polarization in Carpy–Galy Ferroelectrics

In Carpy–Galy compounds, ferroelectricity does not arise from simple *B*‐site off‐centering, but from a complex combination of distortions, predominantly oxygen octahedral rotations and lateral displacements of *A*‐site cations. Symmetry‐mode analysis for La_2_Ti_2_O_7_, shown in Figure 7c, identifies distortion modes (the polar distortion is $\Gamma_{2}^{-}$) that give rise to the ferroelectric $P2_{1}$ cell (Figure 7d). Within the ferroelectric unit cell, two types of *A*‐site La cations can be distinguished: La ions closest to the oxygen spacers (La_c_) exhibit lateral, teardrop‐like doublets, whereas the outer La ions (La_o_) remain nearly undistorted and appear spherical. These two distinct *A*‐site cation environments create alternating zigzag‐like features in HAADF‐STEM images,^[^ ^61^, ^129^ ^]^ with and without teardrop distortions, repeating every two atomic rows along the out‐of‐plane direction (Figure 7e). Notably, the direction of the La_c_ doublet displacements correlates with the in‐plane polarization direction, making them a reliable marker for mapping ferroelectric domains in Carpy–Galy films.^[^ ^61^ ^]^

#### 4.3.3 Mapping Lateral *A*‐Site Cation Off‐Centering in Ruddlesden–Popper Ferroelectrics

The first atomically‐resolved STEM imaging of *A*‐site cation displacements in a Ruddlesden–Popper ferroelectric was performed on Ca_3_Ru_2_O_7_.^[^ ^130^ ^]^ While technically a polar metal (Section 6.1), this material exhibits polar displacements characteristic of hybrid improper ferroelectricity in Ruddlesden–Popper compounds. The annular dark‐field (ADF‐) STEM image in Figure 7f reveals a bilayer structure with alternating lateral displacements of Ca across the film thickness. Lei et al.^[^ ^130^ ^]^ proposed an effective metric to quantify the net in‐plane off‐centering per bilayer, defined as $\Delta u=2\Delta u_{1}-\Delta u_{2}$, where $\Delta u_{1}$ and $\Delta u_{2}$ are the displacements in the two successive perovskite layers (Figure 7f). This method enables direct mapping of in‐plane polarization vectors. Later studies examined atomically‐resolved microscopy of insulating hybrid improper ferroelectrics Li_2_SrNb_2_O_7_,^[^ ^131^ ^]^ (Ca,Sr)_3_Mn_2_O_7_,^[^ ^132^ ^]^ and La_2_SrSc_2_O_7_.^[^ ^133^ ^]^

#### 4.3.4 Unit‐Cell‐Wide Domains in a Dion–Jacobson Film

In Dion–Jacobson films such as CsBiNb_2_O_7_, in‐plane polarization can be mapped through lateral displacements of *A*‐site cations with respect to the alkali spacers. In Figure 7g, Bi^3+^ displacements relative to Cs^+^ columns are used to reconstruct a polarization vector map, revealing 180° ferroelectric domains as narrow as one unit cell. These results, obtained on Dion–Jacobson films by Guo et al.,^[^ ^134^ ^]^ demonstrate the weak interlayer coupling in these ferroelectric phases. Other than mapping ferroelectric domains, HAADF‐STEM was also used to identify Ruddlesden–Popper intergrowths in the Dion–Jacobsons structure.^[^ ^134^ ^]^

### 4.4 Scanning Probe Microscopy

Scanning probe microscopy provides a versatile, multimodal approach to characterize layered ferroelectric films. AFM can provide surface topography information and help identify phase intergrowths or signatures of layer‐by‐layer phase‐pure growth.^[^ ^104^, ^105^, ^118^ ^]^ However, piezoresponse force microscopy (PFM) provides the most important data by granting direct access to in‐plane‐polarized ferroelectric domains. Finally, conductive AFM (c‐AFM) can be used to image charged domain walls abundant in layered ferroelectrics.^[^ ^41^ ^]^

#### 4.4.1 Vector PFM of Uniaxial In‐Plane Polarization

**Figure 8:**
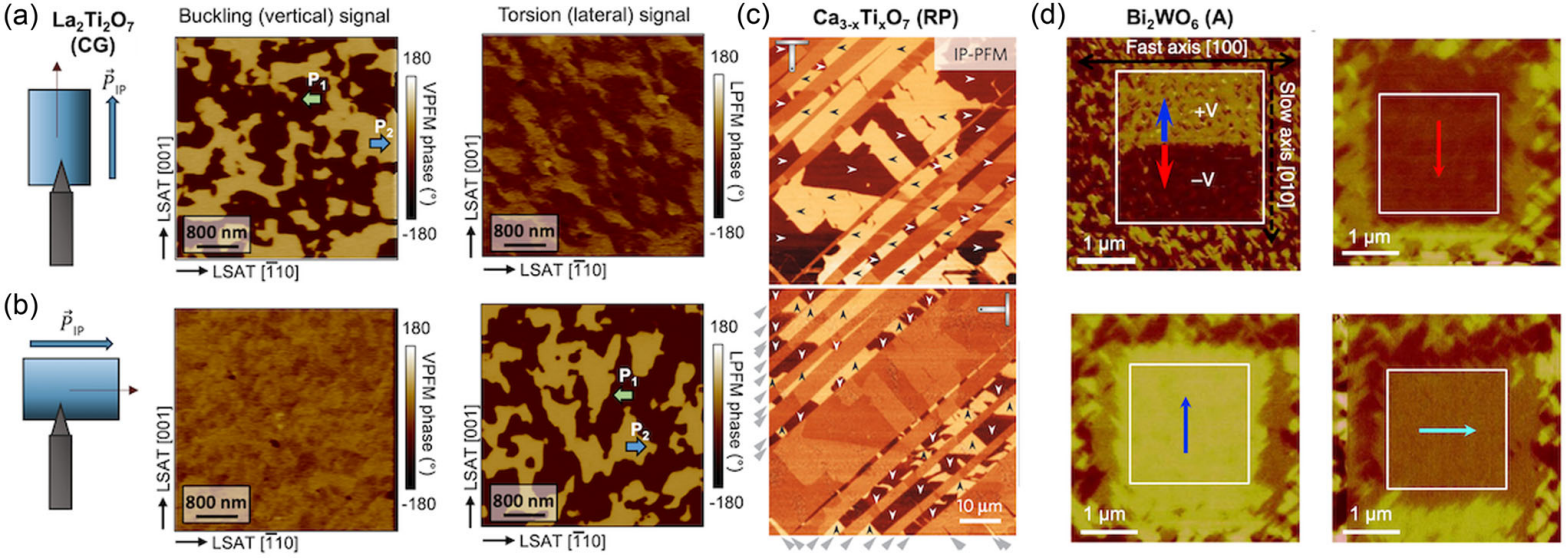
PFM as a probe of in‐plane polarization in layered ferroelectrics. a,b) Vector PFM of a La_2_Ti_2_O_7_ (Carpy–Galy) film.^[^ ^61^ ^]^ With the cantilever parallel to the uniaxial polarization ($P_{\text{IP}}$) the contrast appears in the vertical (buckling) channel; a 90° sample rotation transfers the same domain pattern to the lateral (torsion) channel and leaves the vertical response homogeneous, confirming purely in‐plane polarization. Reproduced with permission.^[^ ^61^ ^]^ Copyright 2025, Wiley‐VCH GmbH. c) Lateral PFM of cleaved Ruddlesden–Popper Ca_2.46_Sr_0.54_Ti_2_O_7_ single crystals.^[^ ^41^ ^]^ The image contains two ferroelastic twin variants whose crystal axes, and hence in‐plane polar axes, are rotated by 90° with respect to each other. When the cantilever is aligned with the polar axis of one variant, that variant shows strong PFM contrast while the other appears nearly featureless. Rotating the sample by 90° swaps the situation, so the previously unresolved crystallographic twin now reveals its 180° ferroelectric domains (white and black arrows) and the contrast within the first variant vanishes. Reproduced with permission.^[^ ^41^ ^]^ Copyright 2015, Springer Nature. d) Trailing‐field‐based switching of a Bi_2_WO_6_ thin film.^[^ ^127^ ^]^ By scanning with ±10–15 V applied to the AFM tip, an in‐plane trailing electric field is generated by the AFM probe. This field reorients the ferroelectric polarization along the slow‐scanning direction, enabling the deterministic writing of 90°‐rotated in‐plane domains. Adapted with permission.^[^ ^127^ ^]^ Copyright 2016, Springer Nature.

In single‐crystal layered ferroelectric films, the polar axis lies entirely in‐plane and is uniaxial.^[^ ^61^, ^105^ ^]^ As shown in **Figure** **8**a,b, aligning the AFM cantilever parallel to this axis produces strong contrast in the vertical (buckling) channel, while a 90° sample rotation shifts the same domain pattern into the lateral (torsion) channel. These complementary measurements upon sample rotation confirm pure in‐plane polarization along a single axis without an out‐of‐plane component^[^ ^61^ ^]^ and therefore single‐crystallinity of the layered ferroelectric film.

#### 4.4.2 Resolving Ferroelastic Twin Variants

Layered perovskites on mismatched substrates (or single crystals) form ferroelastic twins with the polar axes rotated by 90°. In cleaved Ruddlesden–Popper Ca_2.46_Sr_0.54_Ti_2_O_7_ crystals, orienting the cantilever perpendicular to one variant's polar axis yields strong PFM contrast in that twin, while the orthogonal variant shows no contrast (Figure 8c). A 90° sample rotation then reveals the previously hidden 180° in‐plane domains in the second twin, enabling complete mapping of polarization in both crystallographic variants.^[^ ^41^ ^]^

#### 4.4.3 Trailing‐Field Switching of In‐Plane Domains

Even without a bottom electrode, deterministic domain writing is possible via the trailing electric field of an AFM tip.^[^ ^105^, ^135^ ^]^ As illustrated in Figure 8d, scanning with a DC bias of ±10–15 V generates an in‐plane field beneath the tip that aligns the ferroelectric polarization along the slow‐scanning direction. This method allows on‐demand writing of 90°‐rotated in‐plane ferroelastic domains in Auriillius films such as Bi_2_WO_6_.^[^ ^127^ ^]^

#### 4.4.4 c‐AFM for Mapping Conduction at Charged Domain Walls

In oxygen‐deficient Ruddlesden–Popper Ca_2.44_Sr_0.56_Ti_2_O_7–*δ*_ crystals, c‐AFM was employed for mapping charged domain walls in conjunction with lateral‐PFM imaging.^[^ ^41^ ^]^ The polarity‐dependent conductivity was reported: positively charged head‐to‐head walls accumulate *n*‐type carriers and conduct well, whereas negatively charged tail‐to‐tail walls repel carriers and remain insulating.^[^ ^41^ ^]^ Charged domain walls, rare in perovskite ferroelectrics due to their high electrostatic cost, emerge naturally in layered ferroelectrics and can even be engineered through structural defects in thin films (Section 5.3).

### 4.5 Other Complementary Techniques

Several other methods help complete the characterization of layered ferroelectrics. Ferroelectric switching measurements, performed with interdigitated top electrodes to switch the in‐plane polarization,^[^ ^136^ ^]^ are essential for assessing it quantitatively and testing its endurance.^[^ ^61^, ^75^, ^105^ ^]^ To isolate the intrinsic ferroelectric response from leakage contributions, the Positive‐Up Negative‐Down (PUND) method is frequently employed. Optical second‐harmonic generation (SHG) enables domain and domain‐wall imaging;^[^ ^137^ ^]^ additionally, temperature sweeps of the integrated SHG signal yield phase‐transition temperatures.^[^ ^138^ ^]^ Neutron diffraction techniques applied to single crystals of layered ferroelectrics are indispensable for resolving subtle distortions such as oxygen octahedral rotations^[^ ^47^, ^139^, ^140^ ^]^ or incommensurate structural modulations^[^ ^66^ ^]^ that are often invisible in conventional XRD. Finally, dielectric spectroscopy^[^ ^75^, ^79^ ^]^ offers means of probing $T_{C}$ and possible relaxor behavior.

## 5 Uncovering Novel Functionalities in Epitaxial Layered Ferroelectrics

Layered ferroelectrics were first explored mainly as polycrystalline ceramics or bulk single crystals. Only in the past decade has the focus shifted toward thin films (often epitaxial), where advances in growth and characterization now allow us to probe their behavior at the unit‐cell thickness scale. High‐quality epitaxial films with well‐defined polarization orientations have revealed not only the retention of bulk‐like functionalities but also unexpected new phenomena that do not occur in conventional perovskites. In the following, we first discuss how epitaxy enables thin films to retain and, in some cases, even enhance the functional properties known for bulk layered ferroelectrics (Section 5.1). We then highlight the absence of a critical thickness for ferroelectricity in in‐plane‐polarized films (Section 5.2), and describe how the periodic electrostatics of their unit cells, in combination with structural defects, can stabilize unconventional polar textures such as charged domain walls and vortices (Section 5.3). Finally, we outline how the structural compatibility of layered ferroelectrics with perovskites enables new types of hybrid heterostructures and composites (Section 5.4).

### 5.1 Retaining and Enhancing Bulk Properties in Layered Thin Films

Many of the functional properties originally demonstrated in single crystals or polycrystalline ceramics of layered ferroelectrics have successfully translated to the thin‐film geometry, retaining their stability down to just a few unit cells. For instance, the Aurivillius family was historically used for fatigue‐resistant ferroelectric capacitors in the 1990s.^[^ ^30^ ^]^ The exceptional endurance against ferroelectric fatigue has now been confirmed in ultra‐thin films of just 2.5 unit cells, where the ferroelectric response persists for over 10^10^ cycles with a significant in‐plane polarization of 16.5 μC cm^−2^.^[^ ^105^ ^]^

In certain cases, the thin‐film form not only preserves but also significantly enhances the functional properties previously reported in bulk single crystals. For instance, epitaxial stabilization of Carpy–Galy La_2_Ti_2_O_7_ on SrTiO_3_ (110) substrates enabled recording a polarization value of 18.3 μC cm^−2^,^[^ ^61^ ^]^ which is nearly four times higher than previously reported in bulk crystals.^[^ ^59^ ^]^ Simultaneously, the coercive field was substantially reduced, facilitating easier polarization switching. These improvements are attributed to epitaxial control over film orientation, which minimizes crystallographic intergrowths and enforces uniform unit‐cell stacking. Such findings suggest that other overlooked or underestimated compounds of layered ferroelectrics could similarly reveal enhanced functionality when synthesized as high‐quality epitaxial thin films.

Modern thin films of layered ferroelectrics are also considered for new application pathways. For example, the same fatigue‐resistant response that originally made Aurivillius compounds attractive for ferroelectric memory can be re‐engineered and optimized for energy storage performance.^[^ ^141^ ^]^ Here, the materials are leveraged not for their remanent polarization but for their large and recoverable dielectric response.

### 5.2 Absence of Critical Thickness for Ferroelectricity

**Figure 9:**
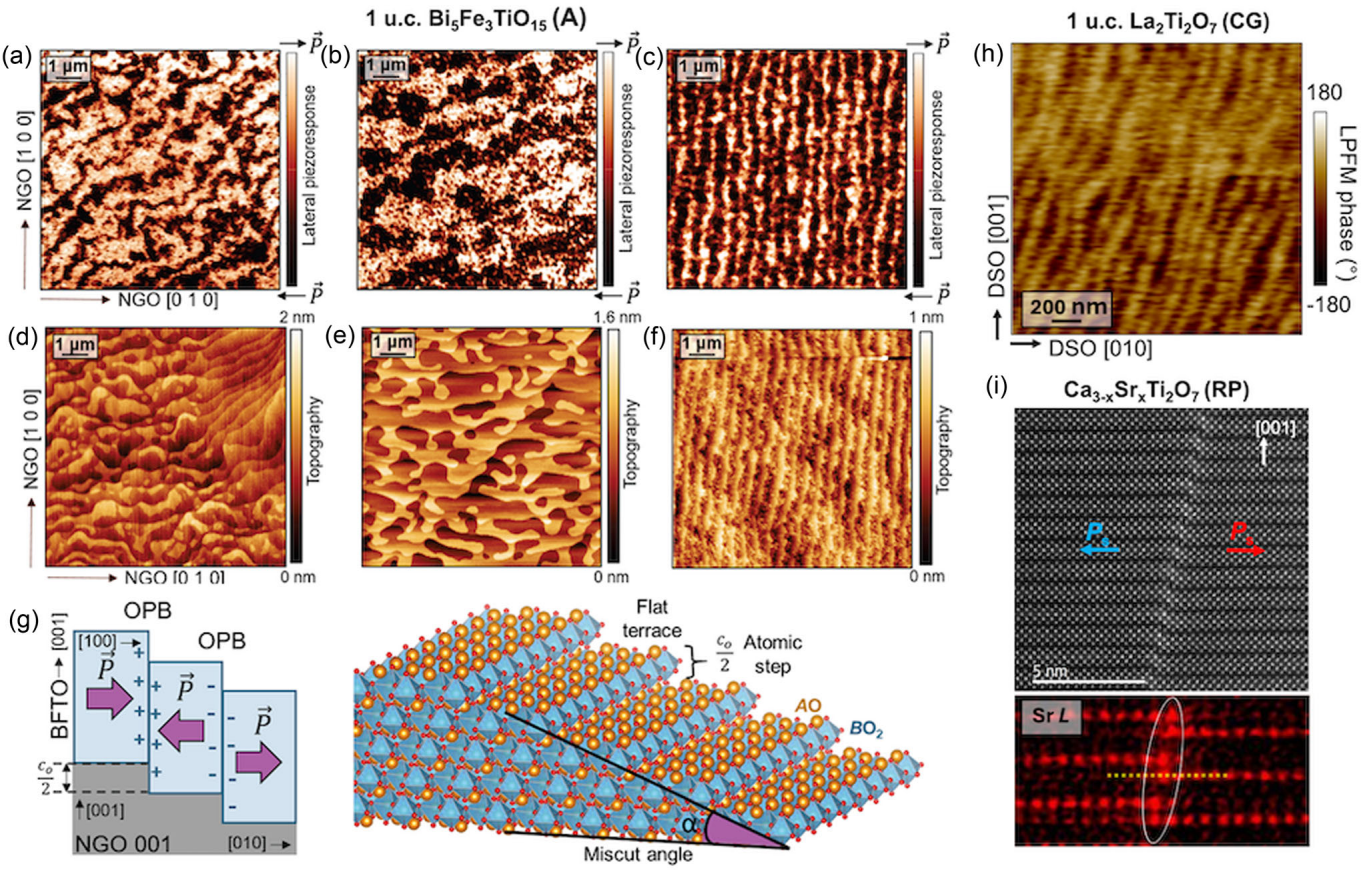
Out‐of‐phase boundaries (OPBs) as nucleation centers for charged ferroelectric domain walls in layered films. a) Irregular striped domain network in a 1 u.c. Bi_5_FeTi_3_O_15_ (BFTO) film corresponding to d) the topography of a small‐miscut (0.02°) untreated substrate. b,e) A wider‐domain pattern in 1 u.c. BFTO layer is achieved after substrate annealing that produces wider terraces. c,f) Highly ordered stripe domains in 1 u.c. BFTO can be produced by a larger miscut (0.05°) substrate with narrowly spaced straight steps. g) Schematics illustrating how every single substrate step in NdGaO_3_ (001) generates a steric OPB in a layered BFTO film.^[^ ^143^ ^]^ It acts as a nucleation site of charged domain walls due to the altered electrostatic and bonding environment and susceptibility to nonstoichiometric defects. Adapted with permission.^[^ ^143^ ^]^ Copyright 2022, American Chemical Society. h) In‐plane PFM image of a 1 u.c.‐thick La_2_Ti_2_O_7_ film^[^ ^61^ ^]^ showing uniform stripe domains, corroborating OPB‐driven domain‐wall formation. Reproduced with permission.^[^ ^61^ ^]^ Copyright 2025, Wiley‐VCH GmbH. i) HAADF‐STEM and energy‐dispersive X‐ray spectroscopy of a charged tail‐to‐tail domain wall coinciding with an OPB in Ca_3−*x*_Sr_x_Ti_2_O_7_^[^ ^150^ ^]^ crystals. Sr segregation at the OPB suggests the mechanism of charge accumulation at such boundaries that can stabilize charged domain walls. Adapted with permission.^[^ ^150^ ^]^ Copyright 2021, Springer Nature.

In‐plane‐polarized ferroelectric films should, in principle, exhibit no critical thickness. Because the polarization lies parallel to the film surface, no bound charge accumulates at the film surface. Therefore, the depolarizing fields that destabilize out‐of‐plane ferroelectrics below four unit cells^[^ ^120^, ^142^ ^]^ are absent. Only very recently, however, thin‐film growth with unit‐cell‐thickness precision enabled by RHEED (Section 4.1) made it possible to test this experimentally. Clear stripe‐domain contrast in lateral PFM was observed starting from just 0.5 unit cell (u.c.) in Aurivillius films^[^ ^104^, ^105^, ^143^ ^]^ (**Figure** **9**a–c) and in 1 unit‐cell‐thick Carpy–Galy films^[^ ^61^ ^]^ (Figure 9h), both grown by PLD. Similar results were obtained for Aurivillius films synthesized by CVD, both in near‐unit‐cell‐thick films^[^ ^144^ ^]^ and in thicker films that were progressively thinned to a few nanometers using an AFM tip to locally remove material.^[^ ^118^ ^]^ The absence of a critical thickness was further confirmed in exfoliated sub‐unit‐cell Aurivillius flakes.^[^ ^145^ ^]^ Dion–Jacobson ferroelectric nanosheets prepared by mechanical exfoliation also exhibit a thickness‐independent piezoresponse,^[^ ^146^ ^]^ reinforcing the generality of this behavior across layered ferroelectrics.

The convergence of evidence from Aurivillius, Carpy–Galy, and Dion–Jacobson systems, prepared by PLD, CVD, and mechanical exfoliation of single crystals, confirms that the absence of a depolarizing field in in‐plane‐polarized layered ferroelectrics is sufficient to eliminate the classical critical thickness. This positions layered ferroelectrics as attractive candidates for ultrathin, energy‐efficient memories, sensors, and actuators.

### 5.3 Nontrivial Polar Textures: Charged Domain Walls and Vortices

Layered ferroelectrics can stabilize polarization configurations that are typically energetically unfavorable in conventional systems. These nontrivial polar textures, such as charged domain walls and vortices, arise due to structural and electrostatic discontinuities introduced by OPBs. As a result, configurations that would be suppressed in standard ferroelectrics can emerge and persist in layered systems. We discuss some examples below.

#### 5.3.1 Charged Domain Walls Nucleated at Out‐Of‐Phase Boundaries

In addition to demonstrating the absence of critical thickness for ferroelectricity in layered in‐plane ferroelectrics, PFM performed on unit‐cell‐thick Aurivillius films revealed unexpected stripe‐like ferroelectric domain patterns^[^ ^105^ ^]^ (Figure 9a–c). Notably, the domain walls run perpendicular to the uniaxial in‐plane polarization of the film, forming a periodic array of alternating charged head‐to‐head and tail‐to‐tail domain walls (Figure 9g). Charged domain walls are promising for domain‐wall nanoelectronics, as they exhibit conduction properties distinct from the surrounding bulk.^[^ ^147^, ^148^, ^149^ ^]^ However, they are typically energetically unfavorable in proper ferroelectrics, implying an unconventional formation mechanism in layered ferroelectrics.

By comparing domain patterns imaged by PFM (Figure 9a–c) with the corresponding substrate topography (Figure 9d–f), a one‐to‐one correlation was established between substrate steps and domain walls.^[^ ^143^ ^]^ Density‐functional theory and phase‐field simulations attribute this to the formation of OPBs:^[^ ^143^ ^]^ when the layered unit cell crosses a step terrace of the substrate, it shifts vertically by a fraction of its height along the out‐of‐plane direction (Figure 9g). This structural discontinuity alters the local electrostatic boundary conditions and is prone to non‐stoichiometries,^[^ ^122^, ^143^ ^]^ which induce and stabilize charged domain walls. Similar OPB‐pinned stripes were later observed in other compositions of Aurivillius films^[^ ^144^ ^]^ as well as in 1‐u.c. La_2_Ti_2_O_7_ (Carpy–Galy) films^[^ ^61^ ^]^ (Figure 9h), again mirroring the underlying substrate terrace morphology. OPBs that host charged domain walls were also observed in Ca_3−*x*_Sr_*x*_Ti_2_O_7_ crystal of Ruddlesden–Popper phase, in which HAADF‐STEM revealed a charged tail‐to‐tail domain wall coincident with an OPB (Figure 9i).^[^ ^150^ ^]^ Energy‐dispersive X‐ray spectroscopy showed local Sr enrichment at the OPB, suggesting the accumulation of ionic charge that compensates the bound negative polarization charge of the wall.

The recurring motif across three different families of layered ferroelectrics suggests that such OPB‐induced charged domain walls are universal for in‐plane‐polarized layered ferroelectrics. This could be explored for precisely engineered locations of charged domain walls and their density by tailoring substrates on which the films grow.

#### 5.3.2 Polar Vortices in Films of Layered Ferroelectrics

**Figure 10:**
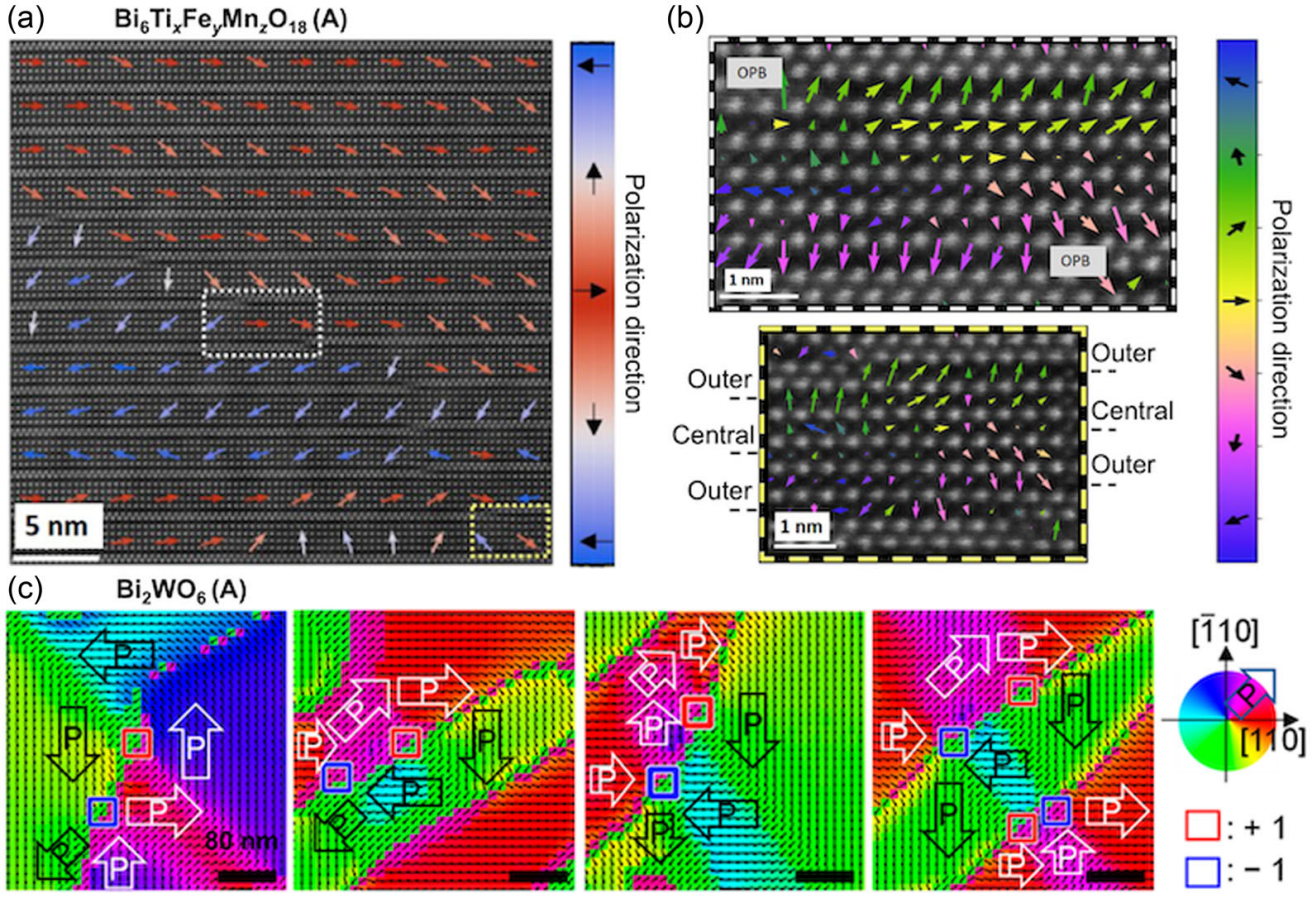
Polar‐vortex textures stabilized in layered ferroelectric thin films. a) Polarization map of a Bi_6_Ti_*x*_Fe_*y*_Mn_*z*_O_18_ Aurivillius film:^[^ ^151^ ^]^ two vortices (dashed boxes) emerge where tail‐to‐tail domain walls intersect OPBs. Adapted with permission.^[^ ^151^ ^]^ Copyright 2022, American Chemical Society. b) Zoomed‐in images of the white and yellow boxes in (a). The OPB breaks the lattice periodicity, generates a strain/electrostatic discontinuity, producing a polarization vortex. c) Angle‐resolved PFM analysis of an epitaxial Bi_2_WO_6_ film.^[^ ^155^ ^]^ Six lateral‐PFM phase maps, acquired at different cantilever orientations, are combined to reconstruct local polarization vectors, revealing flux‐closure vortex structures (red and blue boxes denote winding numbers +1 and −1). The vortices are stabilized by a “hidden” ⟨100⟩‐polarized phase that bridges the four dominant ⟨110⟩ variants. Reproduced with permission.^[^ ^155^ ^]^ Copyright 2023, American Chemical Society.

Moore et al.^[^ ^151^ ^]^ investigated multiferroic^[^ ^152^, ^153^, ^154^ ^]^ Aurivillius Bi_6_Ti_*x*_Fe_*y*_Mn_*z*_O_18_ *n* = 5 films and discovered nanoscale flux‐closure vortices. At OPBs, the film is subject to both a strain gradient and a discontinuity in electrostatic boundary conditions. When a charged domain wall coincides with an OPB—as revealed by atomically resolved STEM polarization vector mapping (**Figure** **10**a), polarization can be forced into a continuous rotation, producing a vortex (Figure 10b). Such a vortex should be charged in the same way as tail‐to‐tail domain walls are, but is expected to have the charge more localized at the vortex core, further increasing local conductivity. For the vortex to form, the lateral distance between successive OPBs must be similar to 180° charged domain‐wall width, which is around 5–8 perovskite cells.^[^ ^151^ ^]^ Because OPB density can be tuned via substrate miscut^[^ ^143^ ^]^ or growth stoichiometry,^[^ ^122^ ^]^ the vortices in Aurivillius films can, in principle, also be engineered.

Vortex‐like polar textures were also uncovered in epitaxial Aurivillius Bi_2_WO_6_ films. Kwon et al.^[^ ^155^ ^]^ applied a high‐resolution angle‐resolved lateral‐PFM protocol, acquiring six lateral‐PFM maps at different cantilever azimuths and reconstructing the full local piezoresponse vector map. This analytical approach uncovered a “hidden” polar phase that links the four conventional polarization variants that appear in flux‐closure vortex domain patterns. The bridge phase enables smooth 90° rotations and organizes polarization variants into flux‐closure vortices with a winding number +1 or −1 (Figure 10c).

### 5.4 Epitaxial Integration of Layered Ferroelectrics with Perovskites

The structural similarity between layered ferroelectrics and conventional perovskites enables their epitaxial integration into heterostructures. Such compatibility opens avenues for engineering artificial materials that combine in‐plane‐polarized layered ferroelectrics with conventional perovskites that can be metallic, magnetic, or out‐of‐plane‐polarized. In this way, one can create novel interfacial phenomena and access functional properties beyond those of the individual phases.

#### 5.4.1 Carpy–Galy and Perovskite Superlattices

**Figure 11:**
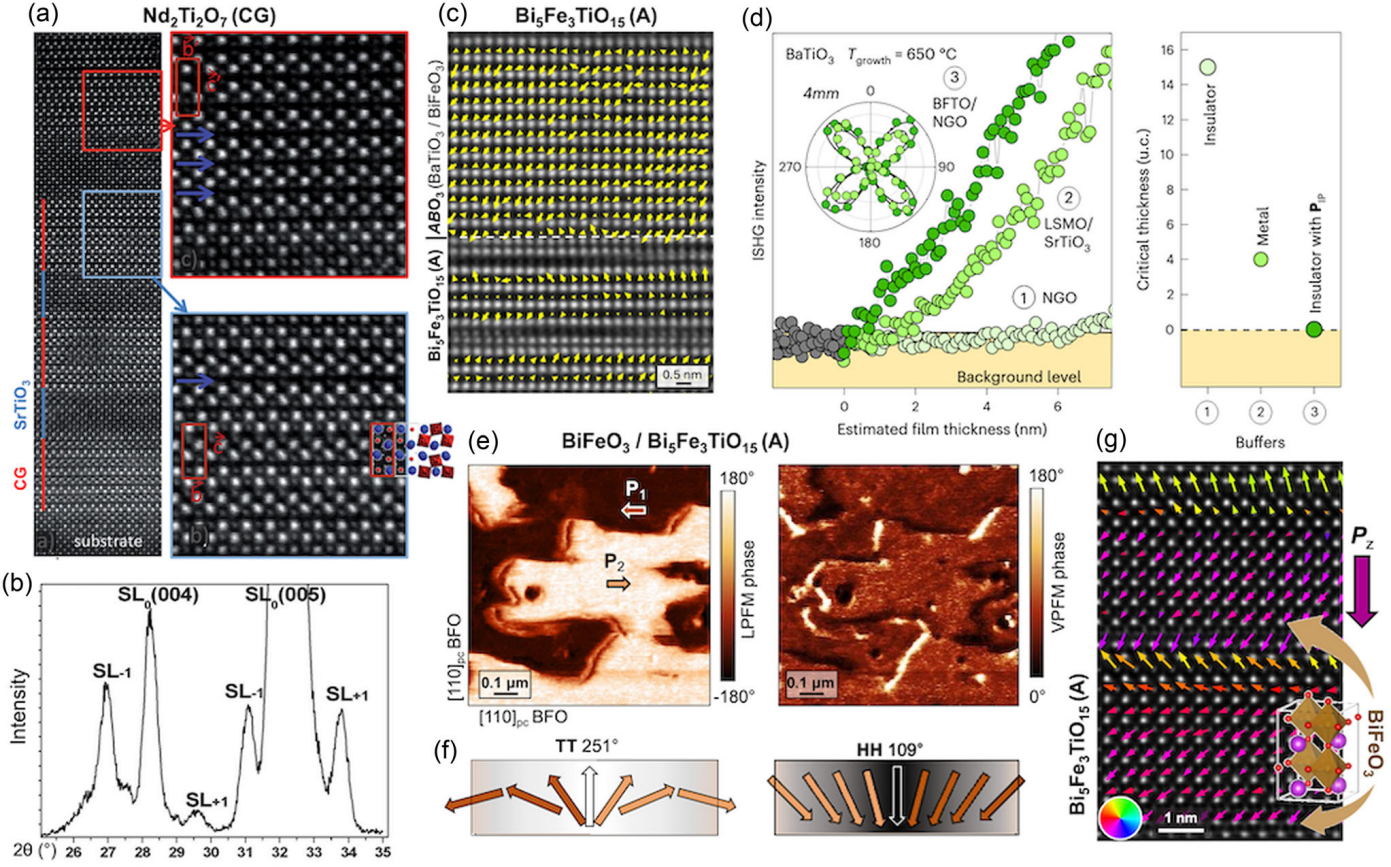
Epitaxial integration of layered ferroelectrics with perovskites. a,b) Coherent stacking of Nd_2_Ti_2_O_7_ (Carpy–Galy) and SrTiO_3_ perovskite blocks in a superlattice.^[^ ^156^ ^]^ (a) The STEM image shows alternating Carpy–Galy (red markers) and STO perovskite (blue markers) layers. Zoom‐in into the blue‐framed region reveals a single kinetically favored metastable *γ* polymorph^[^ ^110^, ^111^ ^]^ stacking fault (arrow), which repeats periodically at higher order (red frame). (b) Superlattice satellite peaks were observed for the two brightest Carpy–Galy XRD reflections. Reprinted with permission.^[^ ^156^ ^]^ Copyright 2018, Royal Society of Chemistry. c–f) Use of in‐plane‐polarized Aurivillius Bi_5_FeTi_3_O_15_ (BFTO) as a buffer for perovskite BaTiO_3_ and BiFeO_3_.^[^ ^53^ ^]^ (c) HAADF–STEM and *B*‐site displacement maps at the BiFeO_3_/BFTO interface demonstrate unit‐cell‐sharp coherence and immediate onset of ferroelectric polarization with no critical thickness. (d) ISHG intensity during BaTiO_3_ growth on (1) bare substrate, (2) metallic electrode, and (3) in‐plane‐polarized BFTO buffer, showing that only the BFTO buffer eliminates the usual critical thickness for ferroelectricity. (e) Lateral and vertical PFM maps of BiFeO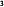 films grown on BFTO reveal two in‐plane polarization variants and a uniform downward out‐of‐plane polarization, with some discontinuities appearing at the domain‐wall contours. These are consistent with ferroelectric domain‐wall homohelicity in BiFeO_3_^[^ ^53^ ^]^ with associated polarization profiles schematically shown in (f). Adapted with permission.^[^ ^53^ ^]^ Copyright 2023, Springer Nature. g) HAADF–STEM overlaid with polarization vectors shows a hybrid order in BiFeO_3_/BFTO composite film, whereby out‐of‐plane dipoles no longer cancel out in the out‐of‐plane direction between the fluorite spacers. This yields a ferrielectric‐like state with a net out‐of‐plane polarization in addition to the in‐plane polarization.^[^ ^54^ ^]^ Adapted with permission.^[^ ^54^ ^]^ Copyright 2025, Springer Nature.

A first demonstration of coherent epitaxy between layered and simple perovskite oxides was achieved in Nd_2_Ti_2_O_7_/SrTiO_3_ superlattices grown by PLD.^[^ ^156^ ^]^ Here, the Carpy–Galy *n* = 4 ferroelectric is epitaxially combined with the cubic SrTiO_3_ perovskite layer.^[^ ^156^ ^]^ HAADF‐STEM reveals a coherently strained superlattice (**Figure** **11**a), that preserves a Carpy–Galy layering (blue box shows a periodic pattern of *A*‐cation zigzags, refer to Section 4.3). A kinetically favored metastable *γ* polymorph^[^ ^110^, ^111^ ^]^ appears as a stacking fault in the layers (marked by a blue arrow) and becomes more frequent with increasing thickness, see the red inset. Superlattice satellites in XRD (Figure 11b) confirm a well‐defined repeat distance and a high quality of such hybrid superlattices between Carpy–Galy and perovskite phases. Local piezoresponse spectroscopy showed that the Nd_2_Ti_2_O_7_ ferroelectric response is attenuated in the superlattice, while current–voltage mapping revealed conductive channels attributed to oxygen‐vacancy tunneling across interfaces.^[^ ^156^ ^]^

#### 5.4.2 In‐Plane‐Polarized Aurivillius Buffer for Out‐Of‐Plane‐Polarized Perovskite Ferroelectrics

Out‐of‐plane ferroelectric thin films typically exhibit a critical thickness of at least 4 unit cells, below which ferroelectricity is suppressed by depolarizing fields.^[^ ^120^, ^142^ ^]^ By integrating an in‐plane‐polarized Aurivillius buffer, which remains ferroelectric down to sub‐unit‐cell thickness^[^ ^105^, ^144^ ^]^ (Section 5.2), it becomes possible to enforce polarization continuity across the interface and stabilize out‐of‐plane polarization from the very first unit cell in BaTiO_3_ and BiFeO_3_ (BFO) films.^[^ ^53^ ^]^ Such heterostructures show an atomically sharp interface between the 1‐u.c.‐thick Aurivillius Bi_5_FeTi_3_O_15_ (BFTO) buffer layer and the perovskite ferroelectric film, see Figure 11c. ISHG measurements reveal the out‐of‐plane polarization onset from the first unit cell when these ferroelectrics are grown on the layered ferroelectric buffer, which is a significant improvement when compared to the critical thickness of 4 and 15 unit cells that is measured for the films grown on a metallic buffer and directly on the substrate, respectively.

When multiferroic BFO is deposited onto a BFTO buffer layer, the in‐plane‐polarized buffer of the Aurivillius phase not only removes the critical thickness for ferroelectricity but also changes its allowed ferroelectric domain and domain‐wall variants.^[^ ^53^, ^157^ ^]^ The BFO film grown on BFTO exhibits two in‐plane polarization variants and a uniform downward out‐of‐plane polarization (Figure 11e). At the domain walls, however, vertical polarization exhibits local discontinuities, implying a continuous rotation of the polarization vector. Specifically, tail‐to‐tail (TT) walls rotate upward, forming 251° domain walls, while head‐to‐head (HH) walls rotate downward across 109°, leading to a defined polarization rotation sense at all walls (Figure 11f). This homohelicity at the domain walls is consistent with a ferroelectric analog to the Dzyaloshinskii–Moriya interaction (DMI)^[^ ^158^ ^]^ and is induced by the Aurivillius buffer layer.^[^ ^53^, ^157^ ^]^

#### 5.4.3 Composite Aurivillius–Perovskite Films

Going beyond epitaxial heterostructures between perovskites and layered ferroelectrics, it was demonstrated that it is even possible to embed perovskite layers directly into the Aurivillius crystal framework using ISHG and RHEED monitoring during the deposition.^[^ ^54^ ^]^ In this way, one can create artificial composite systems with additional perovskite layers of choice sandwiched between the Bi‐rich fluorite‐like spacers. Figure 11g shows a composite film in which BFO unit cells are included within the Aurivillius BFTO *n* = 4 matrix.^[^ ^54^ ^]^ Atomically sharp Bi_2_O_2_ layers are preserved, while allowing Ti and Fe to intermix across perovskite blocks. Polar vector mapping reveals a ferrielectric‐like state along the out‐of‐plane direction: *B*‐site dipoles now form a net out‐of‐plane polarization component. Standard Aurivillius compounds with *n* = even exhibit no net out‐of‐plane polarization, see Figure 7a. However, by inserting additional BFO layers into the framework, the electrostatic balance between the spacer layers is disrupted, and an out‐of‐plane component of polarization, coexisting with the native in‐plane polarization of the layered compound, is stabilized. The inclusion of BFO also controls the magnetic functionality of the composite, as its incorporation induces antiferromagnetic order.^[^ ^54^ ^]^ All this demonstrates that composite films retain the functionality of their Aurivillius and perovskite constituents, offering an exciting pathway toward integrated multifunctional materials.

## 6 Research Opportunities and Application Prospects

The structural architecture of layered ferroelectrics unlocks unique research directions and application prospects. Their polarization is unusually robust to free charge carriers, allowing the coexistence of polar displacements with metallic conductivity and the formation of layer‐confined 2D electron gases (Section 6.1). The same structural motifs facilitate magnetic substitution without suppressing ferroelectricity, while the octahedral tilts and rotations often associated with polarization enable symmetry‐allowed coupling to magnetic order via the Dzyaloshinskii–Moriya interaction. The octahedral tilts and rotations are also expected to support altermagnetic order with polarization‐tunable spin splitting (Section 6.2). Finally, the quasi‐2D crystal framework permits postgrowth soft‐chemistry manipulation, such as exfoliation, ion exchange, and topotactic redox transformations, offering routes to engineer new phases and functionalities beyond as‐grown films (Section 6.3). These features make layered ferroelectrics a practical platform to co‐engineer polarization, spin order, and itinerant carriers within one material scaffold.

### 6.1 Resistance to Charge Doping: Polar Metallicity and 2D Electron Gases

Ferroelectricity and metallicity are traditionally considered incompatible, as free carriers at the Fermi level screen long‐range dipole interactions and hinder the rehybridization processes that enable polar distortions.^[^ ^159^ ^]^ Moreover, ferroelectricity requires reversible switching of polarization under applied electric fields, which was deemed unfeasible in conducting systems. Nonetheless, recent work has established the existence of polar metals, materials that combine polar displacements with metallic conductivity.^[^ ^160^, ^161^, ^162^ ^]^ Theoretical studies suggest that polar displacements can persist if conduction electrons couple weakly to soft phonon modes,^[^ ^163^ ^]^ or can even be enhanced via screening charge rearrangement.^[^ ^164^ ^]^ Polar metallicity was experimentally demonstrated in LiOsO_3_,^[^ ^165^, ^166^ ^]^ while switchable ferroelectricity was achieved in a metallic 2D electron gas hosted at Ca‐doped SrTiO_3_.^[^ ^167^ ^]^ Therefore, it appears that combining ferroelectricity and metallicity is not only possible but also could be useful, as noncentrosymmetric metals exhibit a bulk Rashba effect, allowing for the creation of spin‐polarized currents that potentially could be modulated electrically or by other external stimuli. Below, we examine these concepts in layered perovskite‐based ferroelectrics, where polarization persists under substantial doping and coexists with layer‐confined conduction, as shown by theory and experiment.

#### 6.1.1 Robustness of Polar Displacements in Layered Ferroelectrics upon Doping with Free Charge Carriers

**Figure 12:**
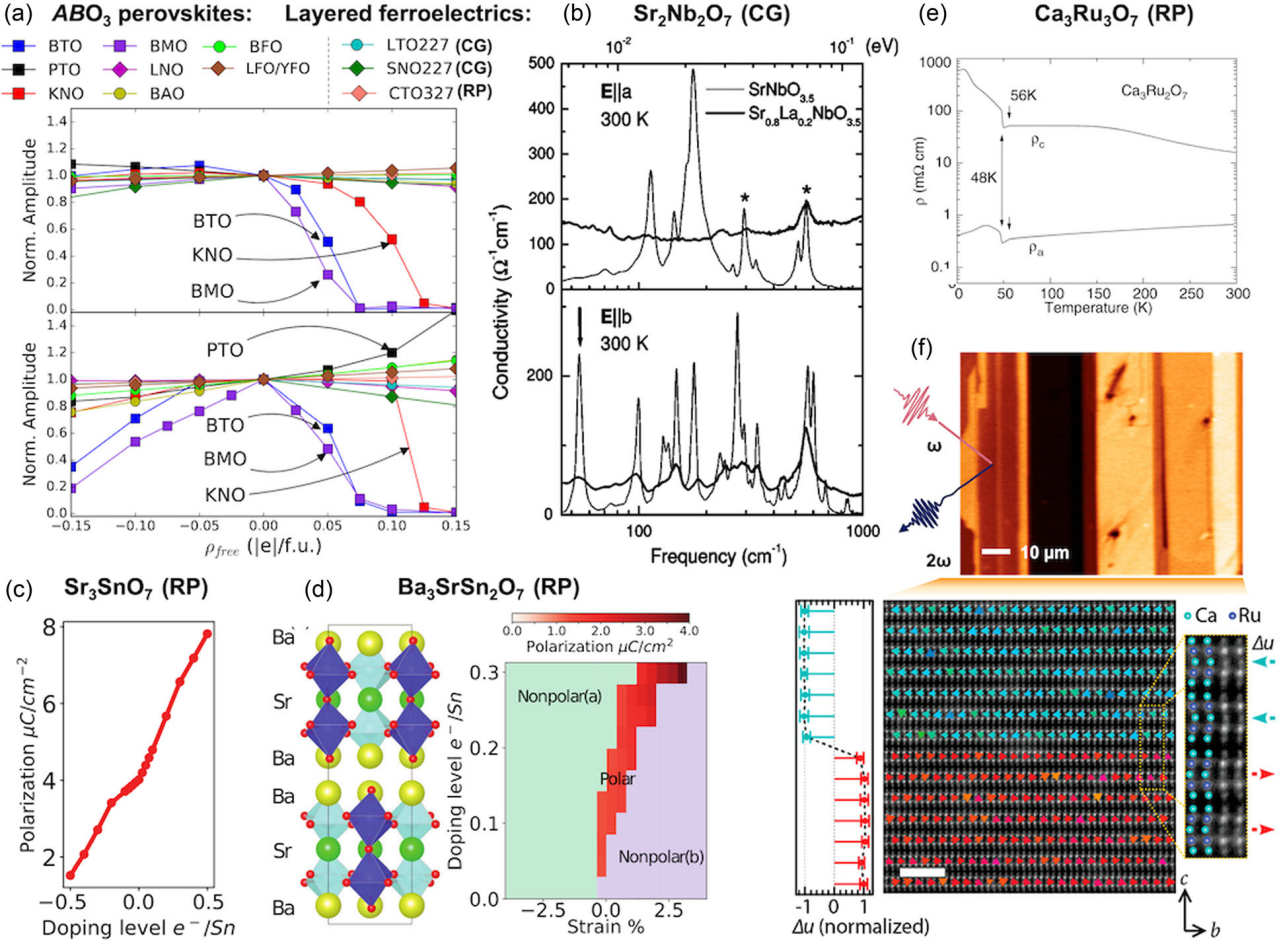
Persistence of polarization in layered ferroelectrics upon free‐carrier doping. a) First‐principles calculations show that in contrast to conventional perovskite ferroelectrics, layered ferroelectrics (e.g., La_2_Ti_2_O_7_ (LTO227), Sr_2_Nb_2_O_7_ (SNO227), Ca_3_Ti_2_O_7_ (CTO327)) retain significant polar displacements even under substantial electron or hole doping.^[^ ^164^ ^]^ Adapted with permission.^[^ ^164^ ^]^ Copyright 2018, American Physical Society. b) Optical conductivity of undoped and La‐doped (equivalent to electron doping) Sr_2_Nb_2_O_7_ reveals persistent phonon modes and the soft mode of the ferroelectric parent phase in the doped compound, suggesting coexistence of metallicity and polar order.^[^ ^170^ ^]^ Reproduced with permission.^[^ ^170^ ^]^ Copyright 2004, American Physical Society. c) In Sr_3_Sn_2_O_7_, polar displacements are theoretically predicted to be enhanced rather than suppressed by electron doping.^[^ ^171^ ^]^ Reproduced with permission.^[^ ^171^ ^]^ Copyright 2021, American Physical Society. d) A doping‐strain phase diagram of Ba_3_SrSn_2_O_7_ shows that electron doping can induce and stabilize polarization in otherwise nonpolar phases.^[^ ^171^ ^]^ Reproduced with permission.^[^ ^171^ ^]^ Copyright 2021, American Physical Society. e) Temperature‐dependent resistivity in Ca_3_Ru_2_O_7_ demonstrates anisotropic metallic behavior.^[^ ^172^ ^]^ Reproduced with permission.^[^ ^172^ ^]^ Copyright 2004, American Physical Society. f) SHG imaging confirms the presence of polar domains, and atomic‐resolution STEM shows a 180° domain wall between two in‐plane polarization directions in Ca_3_Ru_2_O_7_ with measurable off‐centering of Ca ions.^[^ ^130^ ^]^ Adapted with permission.^[^ ^130^ ^]^ Copyright 2018, American Chemical Society.

First‐principles studies have investigated how polar displacements respond to carrier doping in a variety of ferroelectrics.^[^ ^164^ ^]^ While conventional perovskite ferroelectrics such as BaTiO_3_ exhibit a rapid attenuation of polar distortions with increasing electron or hole concentration, several layered ferroelectrics display remarkable resilience (**Figure** **12**a). In particular, La_2_Ti_2_O_7_ and Sr_2_Nb_2_O_7_, both members of the Carpy–Galy family, as well as Ca_3_Ti_2_O_7_, a Ruddlesden–Popper phase, maintain robust polar displacements even under substantial doping levels. Polar metallicity has also been theoretically predicted in related, yet experimentally unrealized Carpy–Galy compounds such as Bi_5_Ti_5_O_17_^[^ ^168^ ^]^ and Bi_5_Mn_5_O_17_.^[^ ^169^ ^]^ These predictions are supported by experimental observations in bulk Sr_2_Nb_2_O_7_,^[^ ^170^ ^]^ where optical conductivity measurements revealed the persistence of phonon modes, characteristic of the insulating ferroelectric phase, even after electron doping via La substitution or oxygen vacancies (Figure 12b). In particular, the ferroelectric soft mode remains detectable in the doped compound, strongly suggesting that polar order coexists with metallic conductivity in these materials. Going one step further, first‐principles calculations suggest that carrier doping can not only preserve but even enhance polar distortions in certain Ruddlesden–Popper phases. For instance, in Sr_3_Sn_2_O_7_, electron doping strengthens the polar displacements^[^ ^171^ ^]^ (Figure 12c), and in Ba_3_SrSn_2_O_7_, doping can even stabilize a polar phase in an otherwise centrosymmetric structure^[^ ^171^ ^]^ (Figure 12d).

#### 6.1.2 Polar Domains Uncovered in a Metal

A different manifestation of polar metallicity is seen in Ca_3_Ru_2_O_7_, a bilayer Ruddlesden–Popper compound long known for its anisotropic metallicity and magnetic ordering.^[^ ^172^ ^]^ Instead of introducing metallicity into a ferroelectric, this case starts from a correlated metal (Figure 12e) and investigates whether polar distortions can coexist with itinerant carriers. Direct evidence for polar order in this compound comes from SHG imaging, which reveals ferroelastic and polar domains at room temperature (Figure 12f). Furthermore, atomic‐resolution STEM measurements uncover in‐plane cation off‐centering consistent with hybrid improper ferroelectricity, and even resolve 180° domain walls separating oppositely polarized regions (Figure 12f).

#### 6.1.3 Why Do Layered Ferroelectrics Tolerate Induced Metallicity?

Several structural and mechanistic factors help explain the robustness of polarization in layered systems, even when free carriers are introduced. First, these compounds often exhibit high Curie temperatures (Section 2.5), which reflects the fact that their ferroelectricity is stabilized not by local *B*–O hybridization as in classical perovskites, but by structural distortions on a larger unit‐cell level. As a result, the ferroelectricity in these materials is inherently more robust against screening from mobile carriers. Second, the underlying polarization mechanisms differ significantly between conventional and layered ferroelectrics. Ruddlesden–Popper phases exhibit hybrid improper ferroelectricity (Section 2.3), which is driven by a coupling between nonpolar modes, while polarization appears only as a secondary effect. Since these primary modes are not directly sensitive to electronic band filling, doping has a limited impact on the polar state. In the case of Carpy–Galy compounds, the situation is more nuanced. These are proper ferroelectrics, but the polarization does not originate from *B*‐site off‐centering, but rather from octahedral rotations and *A*‐site cation displacements (Section 2.2). As this mechanism does not rely on orbital re‐hybridization, it remains largely intact even when *d*‐electrons are introduced through doping.

#### 6.1.4 Creation of 2D Electron Gas in Electron‐Doped Aurivillius Compounds

**Figure 13:**
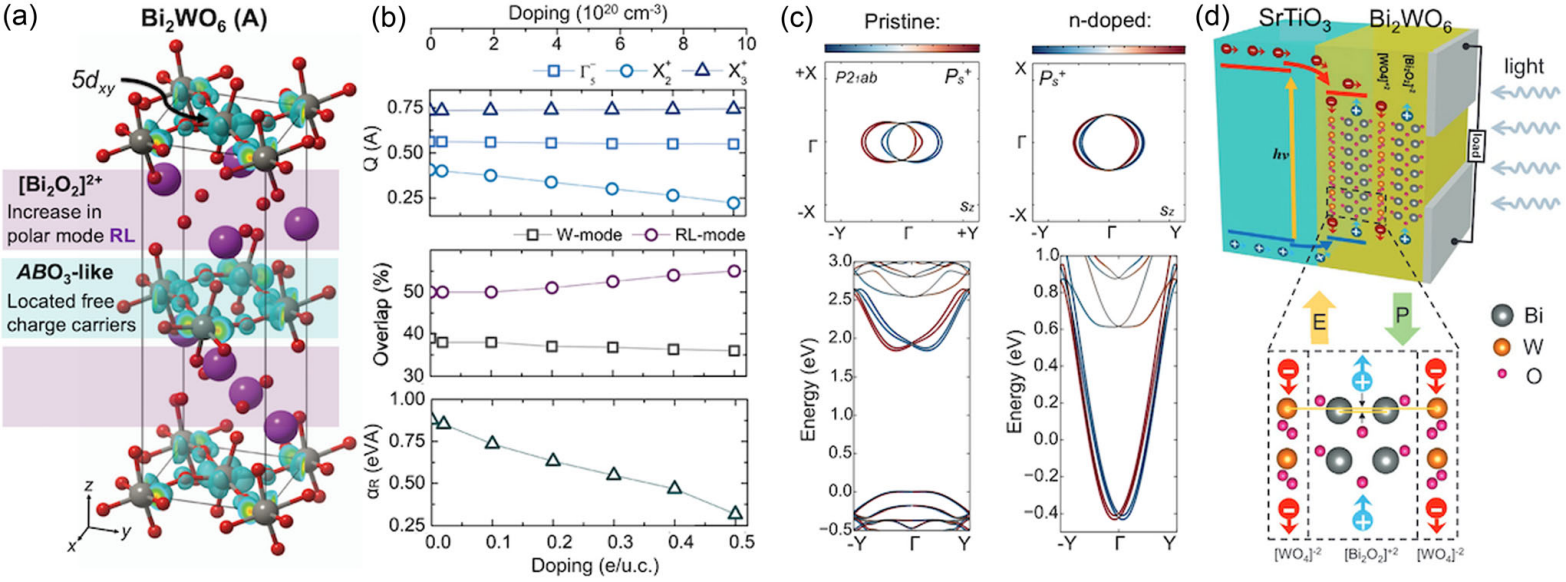
Layer‐selective carrier confinement in electron‐doped Aurivillius ferroelectrics. a–c) Electron doping and Rashba splitting in Aurivillius Bi_2_WO_6_:^[^ ^173^ ^]^ (a) charge‐density of the conduction states in the electron‐doped Bi_2_WO_6_, showing carrier confinement in the perovskite‐like [WO_4_]^2−^ layers, while [Bi_2_O_2_]^2+^ fluorite‐like spacers remain mostly insulating. (b) Evolution of structural distortions as a function of doping reveals that while the total polar mode amplitude remains nearly unchanged, its perovskite‐related component (W‐mode) decreases and Bi_2_O_2_‐related component (RL‐mode) increases, preserving the overall amplitude of the polar mode. The Rashba coefficient decreases with doping but remains finite. (c) Spin‐resolved band structures show retained Rashba splitting in the doped state, opening prospects for ferroelectric Rashba devices. Adapted with permission.^[^ ^173^ ^]^ Copyright 2019, Springer Nature. d) Schematic of photoexcited carrier separation mechanism in Bi_2_O_2_:^[^ ^175^ ^]^ photoexcited electrons from SrTiO_3_ are injected into the perovskite‐like layers of the Aurivillius film, enabling confined transport within a 2D potential defined by the layered structure. The spatial separation of electrons and holes across different layers reduces recombination and results in a giant enhancement in photocurrent compared to perovskite ferroelectrics. Reproduced with permission.^[^ ^175^ ^]^ Copyright 2020, Wiley‐VCH GmbH.

Compared to other layered ferroelectrics, Aurivillius phases have not traditionally been explored in the context of polar metallicity. This is likely due to the fact that their polarization mechanism, characterized by *B*‐cation off‐centering within the perovskite blocks closely resembles that of conventional ferroelectrics. However, recent studies suggest that charge doping in Aurivillius compounds can lead to unusual charge confinement (**Figure** **13**).

Djani et al. demonstrated through first‐principles calculations that in Bi_2_WO_6_, conduction electrons introduced via doping predominantly localize within the perovskite‐like blocks, while the fluorite‐like [Bi_2_O_2_]^2+^ layers remain largely insulating (Figure 13a).^[^ ^173^ ^]^ This spatial separation leads to the formation of a two‐dimensional electron gas (2DEG) within the perovskite layers of the structure, similarly to confined conductivity reported in layered antipolar tungsten bronzes.^[^ ^174^ ^]^ Notably, despite the added carriers, the total polar distortion in Bi_2_WO_6_ remains relatively robust (Figure 13b). By decomposing the polar mode, it was shown that while the displacements in the perovskites diminish (W‐mode), the [Bi_2_O_2_]^2+^ layers become more polar (RL‐mode). Such coexistence of confined conduction and polarization opens up prospects for spintronic applications based on Rashba‐type ferroelectrics, in which spin splitting of the conduction bands can be tuned via an external electric field. Although the splitting parameter decreases with increasing electron concentration (Figure 13b), it remains finite (Figure 13c). These results point to Aurivillius compounds such as Bi_2_WO_6_ as candidates for voltage‐tunable spintronic devices.

#### 6.1.5 Layer‐Confined Carriers Enabling Enhanced Photoresponse

The concept of spatially separated carrier confinement in layered ferroelectrics has also been experimentally harnessed to improve photoconductive performance. In Bi_2_WO_6_/SrTiO_3_ heterostructures, Yang et al.^[^ ^175^ ^]^ demonstrated that photoexcited electrons generated in the SrTiO_3_ substrate are efficiently injected into the Bi_2_WO_6_ conduction bands of the Aurivillius film due to favorable band alignment. Crucially, the layered structure leads to natural confinement: electrons are localized within the perovskite‐like blocks, while holes remain in the fluorite‐like [Bi_2_O_2_] layers (Figure 13d). This spatial separation suppresses electron–hole recombination and extends carrier lifetimes, resulting in an enhancement of the photocurrent. Compared to conventional perovskite ferroelectric BiFeO_3_, the layered Bi_2_WO_6_ heterostructure exhibits orders‐of‐magnitude increase in photoresponse.^[^ ^175^ ^]^ The electronic confinement effects in Bi_2_WO_6_ were also experimentally investigated for negative differential resistance^[^ ^176^ ^]^

### 6.2 Coexistence with Magnetic Order: Multiferroicity and (Alter‐)Magnetoelectric Coupling

The structural and electronic robustness of layered ferroelectrics not only enables coexistence with free charge carriers but also with magnetic ions, opening avenues toward multiferroicity. In many‐layered architectures, the presence of multiple perovskite layers between spacers allows selective magnetic ion substitution without disrupting the overall polar order. Moreover, the low symmetry and complex distortion patterns, often involving octahedral tilts and rotations, can give rise to antisymmetric exchange interactions such as the Dzyaloshinskii–Moriya interaction (DMI). These features facilitate direct coupling between polarization and magnetic order, making layered ferroelectrics exciting candidates for engineering multiferroicity with magnetoelectric coupling^[^ ^7^, ^8^ ^]^ or enabling polarization control of altermagnetic spin splitting.^[^ ^177^, ^178^ ^]^ The sections below detail representative examples showing how polarization can control magnetic order and spin splitting in layered perovskite‐based architectures.

#### 6.2.1 Multiferroic Aurivillius Phases Through Targeted Magnetic Doping

Just as Aurivillius phases served as a foundational platform for exploring ferroelectricity in layered oxides, they also became the subject of early experimental investigations into layered multiferroic thin films. Initial efforts focused on Fe‐doped Aurivillius compounds within the Bi_*n*+1_Fe_*n*−3_Ti_3_O_3*n*+3_ homologous family,^[^ ^49^, ^179^, ^180^, ^181^ ^]^ but the resulting magnetic phases were typically weakly magnetic or antiferromagnetic at low temperatures only. Subsequent doping attempts using cobalt^[^ ^182^ ^]^ or terbium^[^ ^183^ ^]^ showed inconsistent results, often confounded by secondary magnetic impurity phases, highlighting the challenge of controlling phase purity in partial ion substitution.^[^ ^184^, ^185^ ^]^

**Figure 14:**
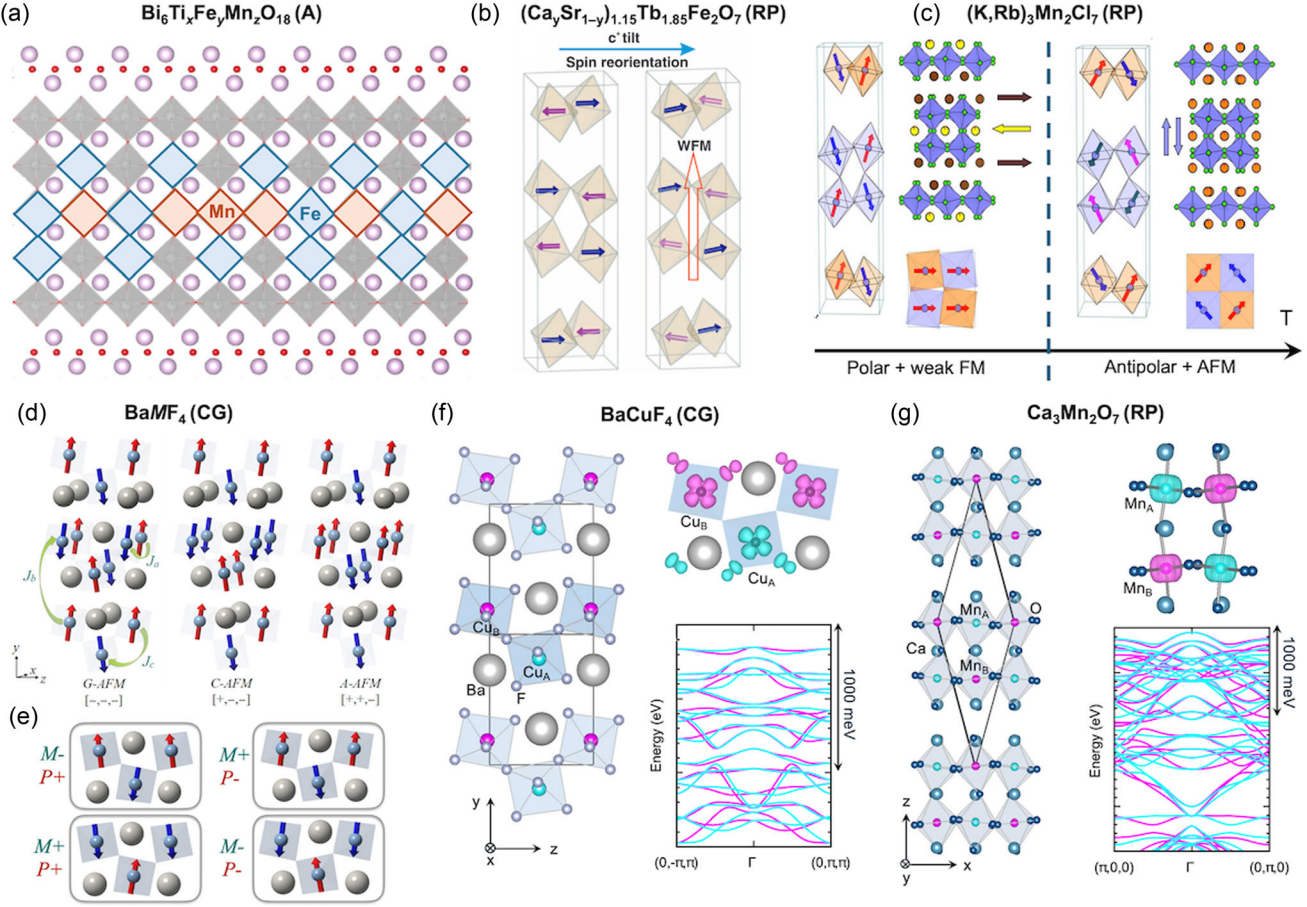
Multiferroic and magnetoelectric phenomena in layered ferroelectrics. a) The *n* = 5 compound Bi_6_Ti_*x*_Fe_*y*_Mn_*z*_O_18_ exhibits room‐temperature ferrimagnetism with magnetoelectric coupling.^[^ ^153^, ^154^ ^]^ Magnetic order is stabilized despite the low doping level through preferential partitioning of Fe and Mn dopants into the central perovskite layers.^[^ ^151^, ^152^, ^187^ ^]^ b) Magnetoelectricity in (Ca,Sr)_1.15_Tb_1.85_Fe_2_O_7_ is achieved by introducing a magnetic *B*‐site sublattice and by using *A*‐site doping to introduce tilts that give rise to spin canting and a weak ferromagnetic moment (wFM).^[^ ^188^ ^]^ Reproduced with permission.^[^ ^188^ ^]^ Copyright 2015, American Association for the Advancement of Science. c) In quasi‐2D halide (K,Rb)_3_Mn_2_Cl_7_, a thermal phase transition converts a polar–wFM state into an antipolar–antiferromagnetic state, enabling simultaneous thermal switching of magnetization and electric polarization.^[^ ^190^ ^]^ Adapted with permission.^[^ ^190^ ^]^ Copyright 2024, Springer Nature. d) Ba*M*F_4_ compounds can adopt three different antiferromagnetic orders, among which either a weak antiferromagnetic moment (G‐AFM)^[^ ^194^ ^]^ or a weak ferromagnetic moment (A‐AFM)^[^ ^197^ ^]^ can emerge via DMI, enabled by the presence of polarization. Adapted with permission.^[^ ^197^ ^]^ Copyright 2018, American Physical Society. e) In BaCuF_4_, which is expected to stabilize in the A‐AFM configuration, four multiferroic states arise from combinations of polarization and magnetization directions.^[^ ^197^ ^]^ Reversing the polarization inverts the octahedral rotations, thereby switching the net magnetization. Adapted with permission.^[^ ^197^ ^]^ Copyright 2018, American Physical Society. f,g) Altermagnetism in (f) BaCuF_4_ and (g) Ca_3_Mn_2_O_7_ nonrelativistic spin splitting associated with altermagnetism is revealed by the magnetization density around the magnetic ions and calculated electronic bandstructure.^[^ ^199^ ^]^ Reproduced with permission,^[^ ^199^ ^]^ arXiv:2411.19928 (2024).

A key advancement came with the cosubstitution of manganese and iron in Aurivillius phases. In particular, the *n* = 5 compound Bi_6_Ti_*x*_Fe_*y*_Mn_z_O_18_ reached magnetic cation concentrations of up to 40%, sufficient to stabilize room‐temperature multiferroicity,^[^ ^153^ ^]^ later proven to be a single‐phase property.^[^ ^186^ ^]^ Magnetoelectric coupling was confirmed via lateral PFM, which revealed modified ferroelectric domain patterns upon application of a magnetic field.^[^ ^153^, ^154^ ^]^ Atomic‐resolution STEM studies^[^ ^152^ ^]^ and first‐principles calculations^[^ ^151^ ^]^ further showed that magnetic cations preferentially occupy the central perovskite layers, driven by elastic and electrostatic interactions. Additionally, increased octahedral tilting and reduced tetragonality in the central layers^[^ ^187^ ^]^ likely support the internal partitioning of magnetic dopants (**Figure** **14**a), enhancing their connectivity and interactions despite relatively low overall magnetic ion concentrations.

#### 6.2.2 Hybrid Improper Ferroelectricity as a Route to Magnetoelectric Multiferroics

The discovery of hybrid improper ferroelectricity provided a mechanistic route to stronger magnetoelectric coupling in single‐phase materials. A combination of nonpolar octahedral rotations and tilts can directly affect magnetic ordering via mechanisms such as DMI. And as more than one lattice distortion can switch the polarization in a hybrid improper ferroelectric, this also means that there are more routes to deterministically control magnetization. Following the initial theoretical proposals,^[^ ^69^ ^]^ one of the first experimental demonstrations of such coupling in a hybrid improper system was reported by Pitcher et al. in 2015.^[^ ^189^ ^]^ By carefully tuning the *A*‐site composition to control the tilt geometry and introducing magnetic Fe on the *B*‐site, they identified a crystal‐chemically engineered multiferroic (Ca,Sr)_1.15_Tb_1.85_Fe_2_O_7_ in the Ruddlesden–Popper phase that remains polar and weakly ferromagnetic above room temperature (Figure 14b), as resolved by neutron powder diffraction. This study showcased how tilt‐mode engineering, enabled by the geometric nature of polarization in hybrid improper ferroelectrics, can drive multiferroicity in layered oxides. A subsequent experimental work focused on Ca_3_Mn_1.9_Ti_0.1_O_7_, another hybrid improper ferroelectric where the optical SHG signal associated with polarization showed a marked enhancement below the Néel temperature,^[^ ^138^ ^]^ indicating strong coupling between spin canting and ferroelectric polarization.

Meanwhile, further exploration of magnetoelectric multiferroicity in hybrid improper ferroelectrics has been extended to other structural families. In Dion–Jacobson phases, a recent study demonstrated ion exchange of Li by Mn in polar Li_2_SrTa_2_O_7_, resulting in MnSrTa_2_O_7_ with coexisting magnetic and ferroelectric order.^[^ ^189^ ^]^ Several theoretical predictions have also expanded the range of candidate materials, including anti‐Ruddlesden–Popper structures, such as Eu_4_Sb_2_O,^[^ ^74^ ^]^ and Ruddlesden–Popper chalcogenides and halides.^[^ ^87^ ^]^ More recently, in a quasi‐2D halide system (K,Rb)_3_Mn_2_Cl_7_, a thermally driven phase transition between a polar weak ferromagnetic state and an antipolar antiferromagnetic state was demonstrated experimentally^[^ ^190^ ^]^ (Figure 14c).

#### 6.2.3 Direct Magnetoelectric Coupling in Carpy–Galy Fluorides

The Ba*M*F_4_ family (*M* = Zn, Mg, Mn, Cu, Ni, Co, Fe) has been among the earliest studied multiferroic fluorides,^[^ ^191^ ^]^ with evidence of linear magnetoelectric coupling.^[^ ^192^ ^]^ Structurally, these compounds belong to the Carpy–Galy family and were investigated soon after the discovery of magnetoelectricity in Cr_2_O_3_.^[^ ^193^ ^]^ They are collinear antiferromagnets and can stabilize in G‐type, C‐type, and A‐type antiferromagnetic orders, with nearly degenerate energies (Figure 14d). Ferroelectricity additionally introduces spin canting along the *c*‐axis via DMI, with distinct outcomes depending on the initial antiferromagnetic order. In G‐AFM order, this spin canting induces a weak antiferromagnetic moment that couples directly to the polarization through octahedral rotations, as predicted for BaNiF_4_.^[^ ^194^ ^]^ The same ordering is adopted in BaCoF_4_, but experiments revealed that anisotropic strain can, in addition, stabilize a weak ferromagnetic (wFM) canting along the *x*‐axis too.^[^ ^195^ ^]^ In contrast, A‐AFM ordering leads directly to a wFM canting along *z* in the presence of DMI, which was experimentally observed in BaCuF_4_.^[^ ^196^ ^]^ Recent first‐principles studies^[^ ^197^ ^]^ predict that in BaCuF_4_, this wFM moment is directly coupled to the polarization via octahedral tilts characteristic of Carpy–Galy structures, which invert upon polarization reversal (Figure 14e). BaCuF_4_ is predicted to exhibit a relatively high Néel temperature (*T*_N_ ≈ 275 K), making it a rare fluoride multiferroic with near‐room‐temperature operation. Unfortunately, currently, there are no known naturally forming Carpy–Galy magnetic oxides that could be explored for such direct magnetoelectric coupling based on proper Carpy–Galy ferroelectricity.

#### 6.2.4 Altermagnetoelectric Coupling in Layered Ferroelectrics

Altermagnets are collinear antiferromagnets that break both time‐reversal and certain crystallographic symmetries, leading to spin splitting in the electronic structure without a net magnetization.^[^ ^178^ ^]^ In layered perovskite‐based oxides, this unconventional order can be stabilized by electronic confinement that drives orbital ordering^[^ ^198^ ^]^ or by structural confinement of octahedral rotation patterns.^[^ ^199^ ^]^ In the layered ferroelectrics discussed in this review, the relationship between tilts and rotations associated with polarization and antiferromagnetism makes them excellent candidates to exhibit altermagnetism. In BaCuF_4_ and Ca_3_Mn_2_O_7_, both of which are layered ferroelectrics, first‐principles calculations reveal *d*‐wave altermagnetic spin order^[^ ^199^ ^]^ (Figure 14f–g). Importantly, octahedral rotations in these materials can be directly controlled by the ferroelectric‐polarization switching, enabling an “altermagnetoelectric effect” in which reversal of the polarization switches the spin‐splitting pattern. This effect provides a mechanism for voltage control of spin currents in antiferromagnets without relying on weak ferromagnetism or relativistic interactions, and opens new directions for spintronic applications in multiferroic materials with layered perovskite‐based architectures.

### 6.3 2D Nature: Exfoliation and Soft‐Chemistry‐Based Phase Transformations

While layered perovskite‐based ferroelectrics are typically treated as 3D solids, their intrinsic structural anisotropy gives them a pronounced 2D character. In hybrid improper ferroelectric (Ca,Sr)_3_Ti_2_O_7_ of the Ruddlesden–Popper family, through neutron scattering experiments, it has been shown that at high temperatures, the perovskite bilayers behave as isolated 2D slabs, with a 3D behavior emerging only at lower temperatures via dipole formation in the Ti–O planes.^[^ ^80^ ^]^ Similar behavior has been reported in Aurivillius phases, particularly those with even *n*, where weak interlayer coupling results in seemingly uncoupled buried in‐plane‐polarized domains across the film thickness.^[^ ^128^ ^]^ These findings indicate that the ferroelectric behavior can reflect a fundamentally 2D‐like character. Beyond electrostatics, the natural layering in these compounds allows for easier mechanical exfoliation along the spacer planes. Aurivillius thin films have been exfoliated using ultrasound to yield ferroelectric flakes of a sub‐unit‐cell thickness.^[^ ^145^ ^]^ More recently, Dion–Jacobson CsPb_2_Nb_3_O_10_ crystals were mechanically exfoliated using adhesive tape, producing few‐monolayer flakes.^[^ ^146^ ^]^ While their interlayer spacing and exfoliation energies are higher than in typical van der Waals materials,^[^ ^146^ ^]^ the process remains accessible. These results highlight a path for producing 2D ferroelectric materials using simple mechanical techniques long established in the van der Waals 2D materials community.

#### 6.3.1 Soft‐Chemistry Phase Transformations, Delamination, and Nanosheet Assembly

**Figure 15:**
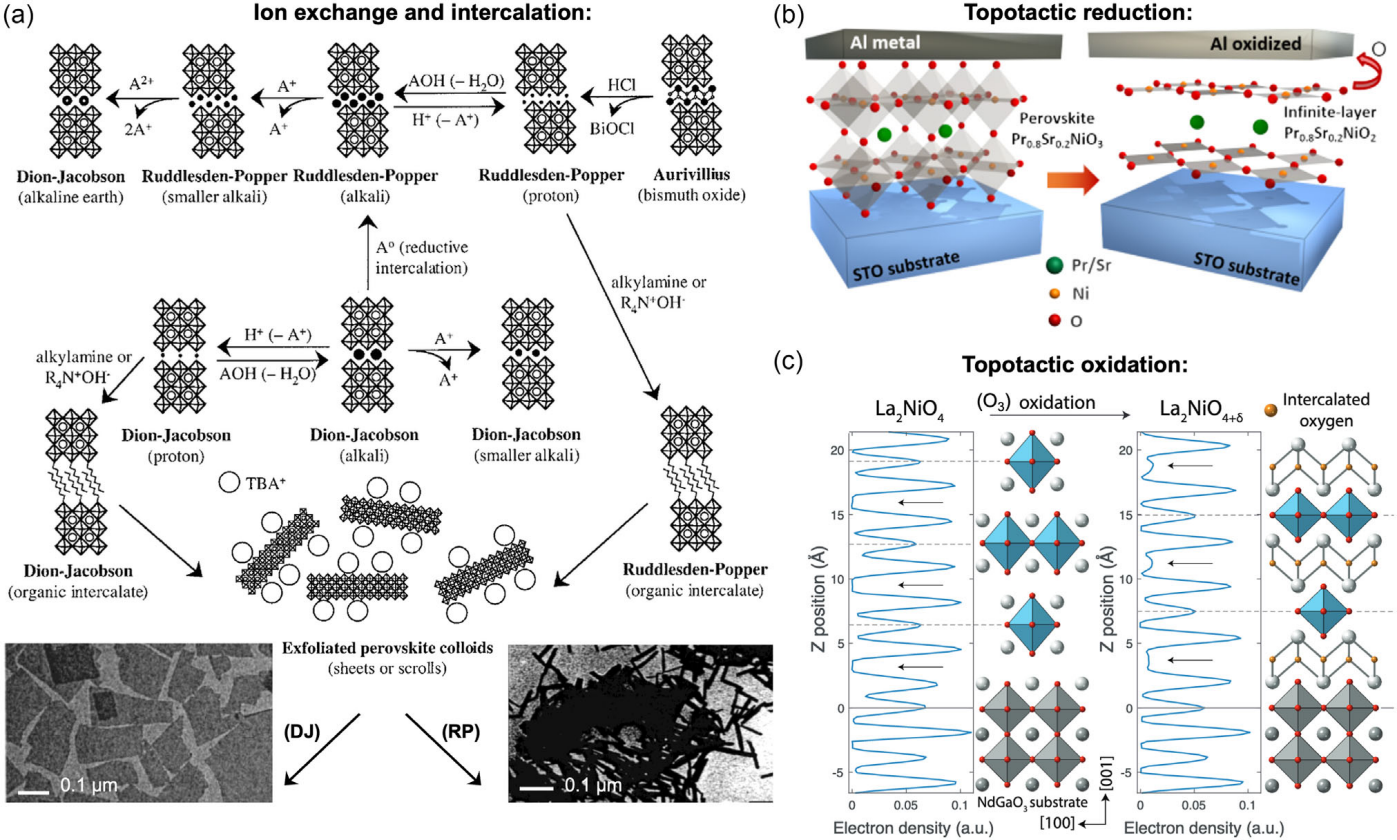
New directions for layered perovskite‐based ferroelectrics through postgrowth solid‐state chemistry approaches. a) Layered perovskites can undergo multiple ion‐exchange and intercalation reactions,^[^ ^200^ ^]^ converting between structural families and enabling colloidal exfoliation of layered perovskite‐based compounds into nanosheets.^[^ ^201^ ^]^ and scrolls.^[^ ^202^ ^]^ Adapted with permission from.^[^ ^200^, ^201^ ^]^ Copyright 2000 and 2002, American Chemical Society. b) Topotactic reduction with an Al overlayer enables transformation of perovskite nickelates into superconducting infinite‐layer phases.^[^ ^206^ ^]^ Reproduced with permission.^[^ ^206^ ^]^ Copyright 2025, Springer Nature. c) Topotactic oxidation of Ruddlesden–Popper nickelates introduces interstitial oxygen into rock‐salt layers, expanding the lattice and creating a new subclass of oxidized layered nickelates with features similar to the Aurivillius family.^[^ ^208^ ^]^ Reproduced with permission,^[^ ^208^ ^]^ arXiv:2506.10262 (2025).

Layered perovskite structures are also ideal candidates for soft‐chemistry reactions, including ion‐exchange, intercalation, and exfoliation through wet‐chemical methods (**Figure** **15**a). We refer the readers to the comprehensive review by Schaak et al.^[^ ^200^ ^]^ for more details. Importantly, Dion–Jacobson and Ruddlesden–Popper compounds can also be delaminated into nanosheets^[^ ^201^ ^]^ or scrolls^[^ ^202^ ^]^ through chemical reactions. This is achieved through protonation of layered oxide phases, followed by treatment with Brønsted bases.^[^ ^81^ ^]^ The resulting nanosheets can be dispersed in colloidal suspensions and restacked via electrostatic self‐assembly into multilayers.^[^ ^201^ ^]^ The restacking process is self‐limiting: absorption halts once surface charges are neutralized, allowing for well‐defined thicknesses and unconventional stacking sequences inaccessible in bulk compounds. Chemical delamination of Aurivillius phases has also been demonstrated,^[^ ^203^ ^]^ although it resulted in Bi from the fluorite‐like spacer layers becoming incorporated into the exfoliated perovskite nanosheets. Once separated into nanosheets, layered perovskites are attractive for applications such as photocatalysis, as they can be further functionalized through co‐catalyst loading, doping, pillaring, etc.^[^ ^204^ ^]^

#### 6.3.2 Blurring the Boundary Between Solid‐State Chemistry Approaches and Physical Vapor Deposition of Thin Films

Originally developed in wet‐chemistry context, such phase transformations through solid‐state chemistry reactions are now being adapted for in situ use during physical thin‐film deposition. In particular, there has been a growing interest in the so‐called topotactic phase transformations,^[^ ^205^ ^]^ which are mediated by the ordered loss/gain and rearrangement of atoms, preserving thin‐film epitaxial orientation. For example, topotactic reduction using a reactive aluminum capping layer allows conversion of perovskite nickelates into infinite‐layer superconductors via oxygen removal and electron doping^[^ ^206^ ^]^ (Figure 15b). This is achieved simply by sputtering a few‐nm‐thick Al layer at elevated temperatures on the perovskite film^[^ ^206^ ^]^ and gives identical results to those achieved through the chemical reduction with CaH_2_.^[^ ^207^ ^]^

Conversely, topotactic postgrowth oxidation with ozone has enabled insertion of interstitial oxygen into Ruddlesden–Popper nickelates, expanding their rock‐salt layers and producing structures closer to Aurivillius‐type phases^[^ ^208^ ^]^ (Figure 15c). These postgrowth modifications are transforming physical deposition processes into a dynamic platform for phase and property engineering. The emerging interplay between physical vapor deposition techniques and soft‐chemistry approaches is expected to bring a shift in how layered perovskite‐based ferroelectrics are designed, offering new possibilities for accessing metastable phases and reversibly tuning functionalities after growth.

## 7 Conclusions and Outlook

Layered perovskite‐based ferroelectrics, once considered structurally cumbersome and technologically outdated, are now reconsidered and are being explored more widely for their unconventional properties in thin films. The natural superlattice structure of these materials was long viewed as a limitation, making them seem difficult to synthesize in high‐quality thin‐film form. However, recent work shows that their intrinsic structural anisotropy can, in fact, aid their growth and provide better orientation control compared to conventional isotropic perovskites. Likewise, their in‐plane polarization was once considered incompatible with standard ferroelectric capacitor architectures. Yet today, with new modes of electrical and optical readout, this characteristic in some cases has become an asset (e.g., absence of critical thickness for ferroelectricity) rather than a hindrance.

This review aimed to present a unified picture of the layered perovskite‐based ferroelectrics, including Aurivillius, Carpy–Galy, Ruddlesden–Popper, and Dion–Jacobson families. By covering developments in thin‐film synthesis, characterization, and newly uncovered functionalities in thin films of these systems, this review highlights how the structurally distinct families of layered ferroelectrics share a surprising number of universal features. These include in‐plane polarization with no critical thickness, high Curie temperatures, and common types of structural defects influencing ferroelectric domain formation. Layered compounds exhibit robust ferroelectricity, epitaxial compatibility with perovskite heterostructures, and nontrivial polar textures. Finally, their intrinsic layering makes them amenable to exfoliation and postgrowth structural modifications through soft‐chemistry approaches.

Recent discoveries in polar metallicity, multiferroicity, and altermagnetic ferroelectrics illustrate how the structural flexibility of layered ferroelectrics could enable novel and exciting effects at the frontier of solid‐state chemistry and condensed matter physics. Their compatibility with spin‐based, optical, and electronic functionalities opens up new directions that extend well beyond classical applications, such as ferroelectric memories. At the same time, short‐term challenges remain, including systematic demonstrations of large‐area, scalable growth and a systematic mapping of which layered compounds are stable in bulk, which ones can be stabilized epitaxially, and how their electric, optical, and ferroelectric properties compare. In this spirit, we hope the review encourages a broader appreciation of layered ferroelectrics, not as isolated material systems, but as a versatile design framework, allowing us to go beyond the properties known for conventional perovskite ferroelectrics.

## Conflict of Interest

The author declares no conflict of interest.
